# Subcutaneous implantable cardioverter-defibrillators: long-term results of the EFFORTLESS study

**DOI:** 10.1093/eurheartj/ehab921

**Published:** 2022-01-28

**Authors:** Pier D Lambiase, Dominic A Theuns, Francis Murgatroyd, Craig Barr, Lars Eckardt, Petr Neuzil, Marcoen Scholten, Margaret Hood, Jȕrgen Kuschyk, Amy J Brisben, Nathan Carter, Timothy M Stivland, Reinoud Knops, Lucas V A Boersma

**Affiliations:** Institute of Cardiovascular Science, University College of London & Barts Heart Centre, West Smithfield, London EC1A 7BE, UK; Department of Cardiology, Erasmus Medical Center, Rotterdam, The Netherlands; Department of Cardiology, King's College Hospital, London, UK; Department of Cardiology, Russells Hall Hospital, Dudley, UK; Department of Cardiology II, University Hospital, Muenster, Germany; Department of Cardiology, Na Homolce Hospital, Prague, Czechia; Thorax Center, Medical Spectrum Twente, Enschede, The Netherlands; Department of Cardiology, Auckland City Hospital, Auckland, New Zealand; Cardiology, Angiology, Hemostaseology and Internal Intensive Care Medicine, Faculty of Medicine Mannheim, University of Heidelberg, Heidelberg, Germany; Rhythm Management Division, Boston Scientific, St Paul, MN, USA; Rhythm Management Division, Boston Scientific, St Paul, MN, USA; Rhythm Management Division, Boston Scientific, St Paul, MN, USA; Department of Cardiology, Amsterdam University Medical Centers, Amsterdam, The Netherlands; Department of Cardiology, Amsterdam University Medical Centers, Amsterdam, The Netherlands; Heart Center, St Antonius Hospital, Nieuwegein, The Netherlands

**Keywords:** Implantable cardioverter-defibrillator, Subcutaneous ICD, Sudden death, Primary prevention, Secondary prevention

## Abstract

**Aims:**

To report 5-year outcomes of EFFORTLESS registry patients with early generation subcutaneous implantable cardioverter-defibrillator (S-ICD) devices.

**Methods and results:**

Kaplan–Meier, trend and multivariable analyses were performed for mortality and late (years 2–5) complications, appropriate shock (AS) and inappropriate shock (IAS) rates. Nine hundred and eighty-four of 994 enrolled patients with diverse diagnoses (28% female, 48 ± 17 years, body mass index 27 ± 6 kg/m^2^, ejection fraction 43 ± 18%) underwent S-ICD implantation. Median follow-up was 5.1 years (interquartile range 4.7–5.5 years). All-cause mortality was 9.3% (95% confidence interval 7.2–11.3%) at 5 years; 703 patients remained in follow-up on study completion, 171 withdrew including 87 (8.8%) with device explanted, and 65 (6.6%) lost to follow-up. Of the explants, only 20 (2.0%) patients needed a transvenous device for pacing indications. First and final shock efficacy for discrete ventricular arrhythmias was consistent at 90% and 98%, respectively, with storm episode final shock efficacy at 95.2%. Time to therapy remained unaltered. Overall 1- and 5-year complication rates were 8.9% and 15.2%, respectively. Early complications did not predict later complications. There were no structural lead failures. Inappropriate shock rates at 1 and 5 years were 8.7% and 16.9%, respectively. Self-terminating inappropriately sensed episodes predicted late IAS. Predictors of late AS included self-terminating appropriately sensed episodes and earlier AS.

**Conclusion:**

In this diverse S-ICD registry population, spontaneous shock efficacy was consistently high over 5 years. Very few patients underwent S-ICD replacement with a transvenous device for pacing indications. Treated and self-terminating arrhythmic episodes predict future shock events, which should encourage more personalized device optimization.

## Structured Graphical Abstract

**Structured Graphical Abstract:**
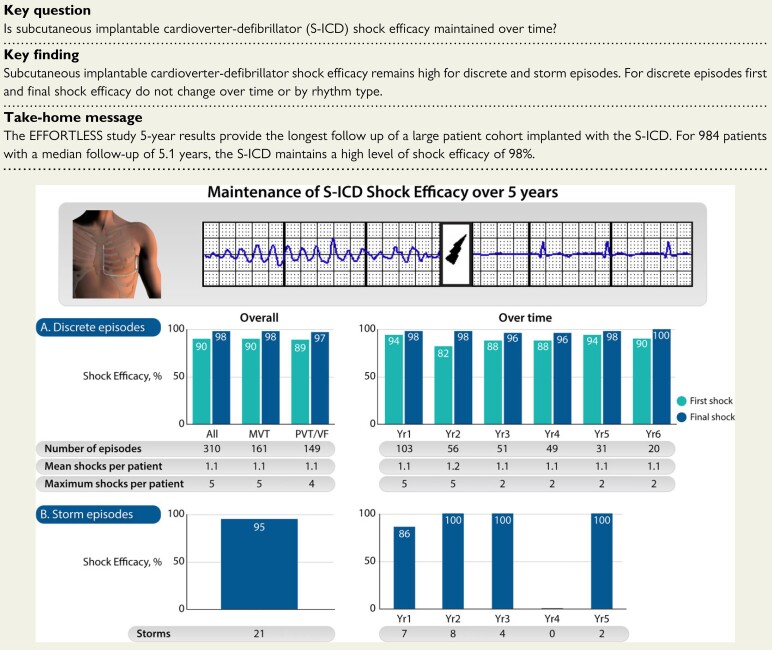
Shock efficacy of appropriate episodes experienced by patients in the EFFORTLESS study. Top left: Torso model of the implant location of the Subcutaneous Implantable Defibrillator (S-ICD) Image provided courtesy of Boston Scientific. © 2022 Boston Scientific Corporation or its affiliates. All rights reserved. Top right: Example S-ICD electrogram recording of a shock (lightning bolt icon) delivered to convert a ventricular arrhythmia. (A) Discrete episodes. (B) Storm episodes.

**See the editorial comment for this article ‘Long-term results of first-generation S-ICD systems: strong from the start and through the threats of time’, by Riccardo Cappato, https://doi.org/10.1093/eurheartj/ehac137.**

## Introduction

Subcutaneous implantable cardioverter-defibrillator (S-ICD) technology had a significant impact on device selection for patients not requiring bradycardia, resynchronization, or anti-tachycardia pacing (ATP), enabling the avoidance of transvenous leads. However, with the introduction of new technologies, there is an imperative to monitor long-term outcomes, making adaptations necessary to optimize therapy. While early complications and efficacy of S-ICD therapy have been reported, limited data are available on later events. The EFFORTLESS registry was designed to systematically track outcomes over 5 years, with the opportunity to examine a wide spectrum of patients in an open-label, real-world study. One- and 3-year outcomes have been reported.^1,2^ Here, we describe the final 5-year results focusing on (i) late complications, (ii) inappropriate shock (IAS) and appropriate shock (AS) rates, (iii) shock efficacy, (iv) defibrillation testing (DFT) on generator replacement, and (v) mortality, along with specific analysis to understand the predictors of later events.

## Methods

### Registry design

The EFFORTLESS S-ICD registry^3^ is an observational, non-randomized, standard of care registry that enrolled 994 patients at 46 centres in 11 countries (Czech Republic, Denmark, France, Germany, Ireland, Italy, the Netherlands, New Zealand, Portugal, Spain, and the UK) from February 2011 to November 2014. The Registry is conducted according to the Helsinki Declaration and ISO 14155:2009 and registered on ClinicalTrials.gov (NCT01085435). All patients provided informed consent according to national and institutional regulations. Patients were followed as per institutional standards up to 60 months post-implant. All scheduled and unscheduled follow-ups for the first-year post-implant were recorded, while in years 2–5 post-implant there was a minimum annual follow-up data requirement (including all adverse events, spontaneous arrhythmia episodes, and programming changes). Patients were enrolled prospectively and retrospectively. Specific contraindications included indications for bradycardia pacing and cardiac resynchronization therapy, presence of ventricular tachycardia amenable for termination by ATP. Subcutaneous implantable cardioverter-defibrillator device programming was set at the investigator’s discretion.

Pre-specified endpoints were perioperative (30 days post-implantation) S-ICD complication rate, 360-day S-ICD complication rate, and the percentage of IAS for AF or supraventricular tachycardia (SVT). Appropriate, inappropriate spontaneous episode, and adverse event classification were performed as described previously.^1^ Specifically, complications were defined as adverse events that resulted in invasive intervention. The EFFORTLESS registry protocol defined complications types as: type I, caused by the S-ICD system; type II, caused by the S-ICD system’s user manual or labelling of the S-ICD system; type III, not caused by the S-ICD system, but would not have occurred in its absence.^3^ Subcutaneous implantable cardioverter-defibrillator device complications refer to type I complications and S-ICD system- and procedure-related complications refer to types I–III complications.

During the study, field advisories were issued for a subset of model 1010 devices,^1^ while three field advisories were issued in December 2020 for EMBLEM devices and model 3501 electrodes.^4^ Device changes were performed throughout the study based on the regular elective replacement indicator.

### Statistical analysis

Descriptive statistics are reported using mean ± standard deviation or median (interquartile range, IQR) for continuous variables and frequency and percentage for categorical variables. Variables were compared using *χ* ^2^ test. Kaplan–Meier analyses were used to estimate event-free rates for complications, IAS, AS, mortality, and events in years 2–5 (complications and appropriate/inappropriate therapy). Univariable and multivariable analysis methods are described in Supplementary material online, Methods. All statistical analyses were performed using SAS version 9.4 (SAS Institute, Cary, NC, USA).

## Results

### Patient characteristics and disposition

Overall, 994 patients were enrolled and 984 S-ICDs were implanted. The EFFORTLESS study enrolment period began on 2 February 2011 and the last study follow-up was completed on 7 July 2020, with the median implant duration being 5.1 years (IQR 4.7–5.5 years) across the patient population. Patient demographics are shown in Supplementary material online, *Table S1*. The mean age of patients at implant was 48 ± 17 years, the majority were male, and the mean left ventricular ejection fraction (LVEF) was 43.4 ± 18.2% (range 9–86%). Almost half had ischaemic (29%) or dilated (18%) cardiomyopathy, while 20% had channelopathies. Most study patients (65%) had a primary prevention indication, including 66% and 91% of the patients with arrhythmogenic right ventricular (ARVC) and hypertrophic cardiomyopathy (HCM) diagnoses, respectively. Of the 593 primary prevention patients, 303 (51%) had LVEF ≤ 35% (aged 58 ± 14 years). One hundred thirty-nine patients had been previously implanted with a transvenous ICD (TV-ICD) system and 30 patients with a pacemaker.

**Figure 1:**
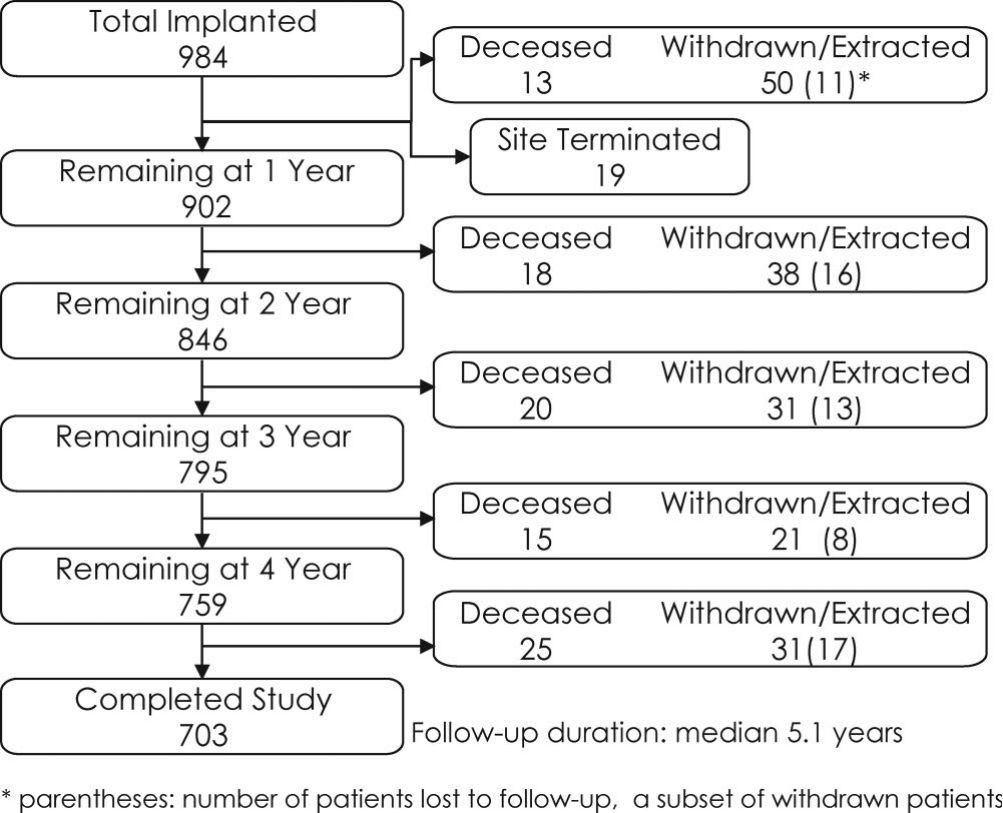
Patient flow chart for the EFFORTLESS subcutaneous implantable cardioverter-defibrillator registry.

Patient disposition throughout the study is shown in *Figure 1*. Over 70% of patients enrolled completed the study; 9.2% patients died, 171 withdrew including 87 (8.8%) who were explanted and 65 (6.6%) patients lost to follow-up; and 19 patients were from a site that terminated study participation. Reasons for study exit are provided in Supplementary material online, *Table S2*. Reasons for device extraction during the follow-up period included infection (*n* = 25), need for pacing (*n* = 20), IASs (*n* = 12), erosion (*n* = 9), and discomfort (*n* = 6). Pacing needs for the patients (2.0%) requiring S-ICD replacement for pacing indications were four for bradycardia, seven for ATP, and nine for cardiac resynchronization therapy.

#### Generators and leads implanted

Generation (Gen) 1 pulse generators (model 1010) and model 3010 electrodes were initially implanted in all patients. During the study, 71/984 patients (7.2%) underwent a generator replacement, mostly for battery depletion (*n* = 42) or system infection or erosion or both (*n* = 15) (Supplementary material online, *Table S3a*) while 15/984 patients (1.5%) underwent electrode replacement, mostly for infection (*n* = 9), noise (*n* = 1), and suspected electrode damage at implant (*n* = 2) (Supplementary material online, *Table S3b*). Overall, including device replacements, patients in the EFFORTLESS registry were implanted with 1010 (95.4%) Gen 1 (model 1010), 27 (2.6%) Gen 2 (model A209), and 21 (2.0%) Gen 3 (model A219) pulse generators and 994 (99.6%) model 3010, 2 (0.2%) model 3401, and 2 (0.2%) model 3501 electrodes. While the SMART Pass filter was not initially available for Gen 2 devices, it became available after April 2016 but was not tracked in this registry. There were 47 patients with a SMART Pass capable device after generator replacement.

### Complications

The registry population was divided into year 1 and years 2–5 to investigate differences in burden and the factors affecting early and late events. *Table 1* summarizes complications by year and Supplementary material online, *Table S4* summarizes complications by year and gender. There were no cases of endocarditis types I–III complications.

**Table 1:** Complications, overall, year 1, and years 2–5

Complication term	Overall (*n* = 984)		Year 1 (*n* = 984)		Years 2–5, all (*n* = 912)		Years 2–5, complication in year 1 (*n* = 56)		Years 2–5, no complication in year 1 (*n* = 856)	
	Events	Subjects	Events	Subjects	Events	Subjects	Events	Subjects	Events	Subjects
		*n* (%)		*n* (%)		*n* (%)		*n* (%)		*n* (%)
Infection requiring device removal	34	31 (3.2)	26	25 (2.5)	8	8 (0.9)	2	2 (3.6)	6	6 (0.7)
Erosion	23	23 (2.3)	10	10 (1.0)	13	13 (1.4)	4	4 (7.1)	9	9 (1.1)
Inappropriate shock requiring intervention: cardiac oversensing	17	16 (1.6)	5	5 (0.5)	12	11 (1.2)	0	0 (0.0)	12	11 (1.3)
Other procedural complications	12	12 (1.2)	7	7 (0.7)	5	5 (0.5)	0	0 (0.0)	5	5 (0.6)
Discomfort	11	11 (1.1)	3	3 (0.3)	8	8 (0.9)	2	2 (3.6)	6	6 (0.7)
Haematoma	9	9 (0.9)	8	8 (0.8)	1	1 (0.1)	0	0 (0.0)	1	1 (0.1)
PG movement	8	6 (0.6)	5	3 (0.3)	3	3 (0.3)	0	0 (0.0)	3	3 (0.4)
Premature cell battery depletion	8	8 (0.8)	2	2 (0.2)	6	6 (0.7)	0	0 (0.0)	6	6 (0.7)
Sub-optimal electrode position	8	8 (0.8)	8	8 (0.8)	0	0 (0.0)	0	0 (0.0)	0	0 (0.0)
Electrode movement	7	7 (0.7)	6	6 (0.6)	1	1 (0.1)	0	0 (0.0)	1	1 (0.1)
Incision/superficial infection	6	6 (0.6)	5	5 (0.5)	1	1 (0.1)	0	0 (0.0)	1	1 (0.1)
Unable to convert: during procedure	5	5 (0.5)	4	4 (0.4)	1	1 (0.1)	0	0 (0.0)	1	1 (0.1)
Inappropriate shock requiring intervention: non-cardiac oversensing	4	4 (0.4)	2	2 (0.2)	2	2 (0.2)	0	0 (0.0)	2	2 (0.2)
Inappropriate shock requiring intervention: SVT above discrimination zone (normal device function)	4	4 (0.4)	1	1 (0.1)	3	3 (0.3)	0	0 (0.0)	3	3 (0.4)
Other technical complications	4	4 (0.4)	1	1 (0.1)	3	3 (0.3)	0	0 (0.0)	3	3 (0.4)
Inability to communicate with the device	3	3 (0.3)	0	0 (0.0)	3	3 (0.3)	0	0 (0.0)	3	3 (0.4)
Sub-optimal PG and electrode position	3	3 (0.3)	3	3 (0.3)	0	0 (0.0)	0	0 (0.0)	0	0 (0.0)
Sub-optimal PG position	1	1 (0.1)	1	1 (0.1)	0	0 (0.0)	0	0 (0.0)	0	0 (0.0)
Total	167	141 (14.3)	97	87 (8.8)	70	60 (6.6)	8	6 (10.7)	62	54 (6.3)

PG, pulse generator; SVT, supraventricular tachycardia.

**Figure 2:**
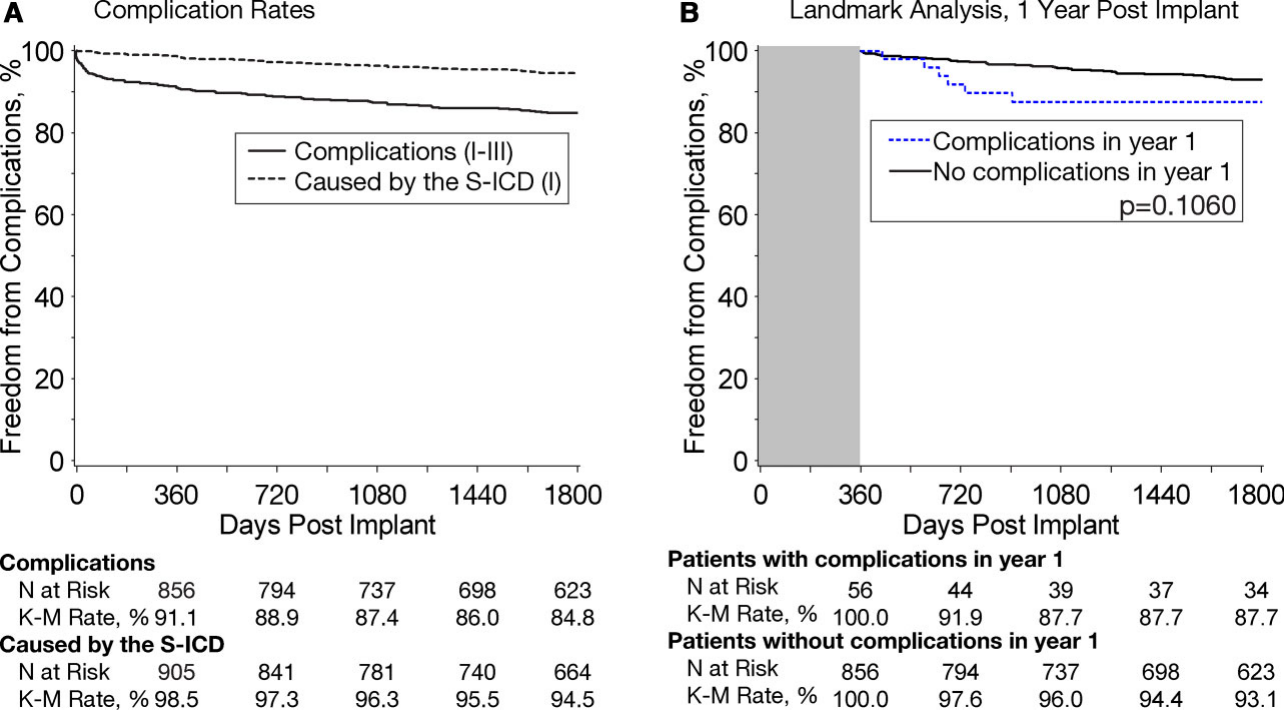
(*A*) Kaplan–Meier complication-free rates for overall complications (types I–III) and for complications caused by the subcutaneous implantable cardioverter-defibrillator system (type I). (*B*) Kaplan–Meier complication-free rates in years 2–5 comparing patients who did and did not experience a complication in year 1.

The S-ICD complication-free rate was 99.9% at 30 days, 98.5% at 360 days,^1^ and 94.5% after 5 years (*Figure 2A*). The main system complications between years 2 and 5 were IAS requiring intervention, discomfort, and premature cell battery depletion (*Table 1*).

The incidence of system or procedure-related complications was 5.6% at 60 days, 8.9% at year 1, and 15.2% at 5 years (*Figure 2A*). The most common complications in year 1 and years 2–5 were infection and erosion requiring system removal (*Table 1*). System infection site was available for 30/34 cases: 23 at the S-ICD pocket, four at the upper electrode incision, one at the pocket and upper incision, two at the pocket and electrode (electrode location not specified). Patients with a previous TV-ICD extracted due to infection (73 or 7.4%) had 3.6% and 3.0% survival rates of experiencing an infection or erosion, respectively, compared with other patients in the study with a 3.5% risk of infection (*P* = 0.86) and a 2.5% risk of erosion (*P* = 0.26).

All implanting physicians were informed and advised of their EFFORTLESS patients with pulse generators and leads related to the advisories issued over the study period. Of system-related complications, none was related to electrode overstress due to variations in header assembly, electrode body fracture, or memory corruption. There were eight (0.8%) early battery depletions. No complications were related to the recent advisories affecting the 3501 model lead and low-voltage capacitor impacting battery depletion in Gen 2 and Gen 3 devices.^4^ One patient developed noise on their lead requiring replacement at 3.5 years due to suspected lead failure, which was not confirmed on the investigation.

A total of 71 patients underwent generator replacement; the median time from original implant to replacement was 4.4 years (IQR 2.3–4.9 years). All except 10 of these patients had device extraction and generator replacement taking place during the same procedure; for the remaining 10 patients, the median time from extraction to replacement was 91 days (IQR 60–164 days). Only one of these patients experienced infection that required system removal during device replacement. Of the 31 subjects with systemic infection, an S-ICD was implanted in eight (23.5%) cases. Of the 23 erosions, 10 (43.5%) had an S-ICD. The remaining patients withdrew from the study before device re-implant; it is assumed that these patients received a TV-ICD.

For the 42 patients who had device replacements due to battery depletion, median time from implant to replacement was 4.8 years (IQR 4.3–5 years) and the median remaining battery life was 23% (IQR 9–34). The majority of patients are yet to have generator replacement: for patients who completed the study with their originally implanted devices, the remaining battery life was available for 597 patients (median 18%, IQR 12–24%).

#### Timing of and factors determining late complications

Only 6 of the 87 (6.9%) patients who experienced a complication in year 1 also had a complication of infection, erosion, and discomfort in years 2–5 (*Table 1*). Patients who had a complication in year 1 were not more likely to have a complication in years 2–5 (*Figure 2B*). Supplementary material online, *Table S5* and *Figure S1* describe univariable and multivariable predictors of one or more complications over years 2–5, respectively, with the main multivariable predictors being no conversion testing performed within 30 days of implant, prior cardiac arrest, history of AF, and history of valve surgery. Importantly, complications in year 1 did not predict later complications. Female gender was a significant univariable but not multivariable predictor of complications in years 2–5.

### Inappropriate shocks

**Figure 3:**
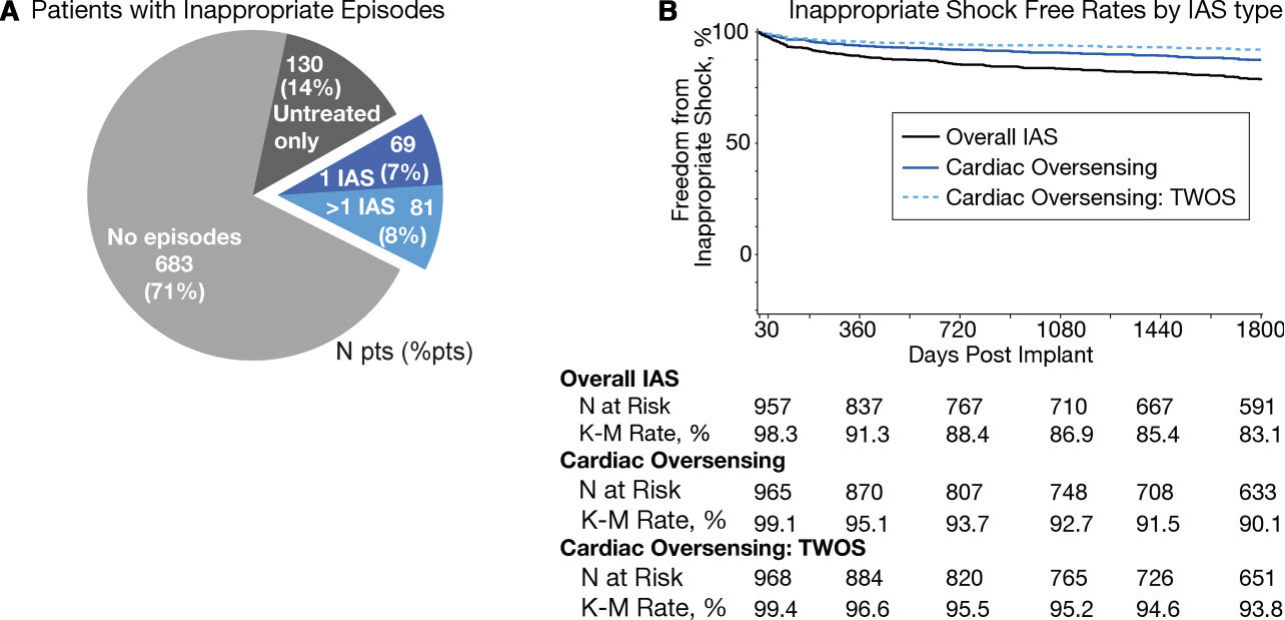
(*A*) Pie chart of patients’ experience with inappropriate episodes; number of patients and percent of patients provided. (*B*) Kaplan–Meier inappropriate shock-free rates for all inappropriate shock, for inappropriate shock due to cardiac oversensing, and for inappropriate shock due to cardiac oversensing of T-waves.

*Figure 3A* describes the incidence of IAS: 7% of patients experienced one episode and 8% had >1 episode. The Kaplan–Meier IAS rate was 16.9% at 5 years (*Figure 3B*). Of the 83 patients who had IAS in the 1st year, one-fifth (21.7%) experienced subsequent IAS.

Altogether, 155 patients experienced 328 IAS episodes during the EFFORTLESS study. The most common cause of IAS was cardiac oversensing in 106 patients (68.3%), followed by IAS for AF/SVT (18.7%) and IAS for non-cardiac oversensing (16.8%).

The relative occurrence of causes of IAS did not differ significantly between year 1 and years 2–5, except for cardiac oversensing IAS due to low amplitude signals (64.7% *P* = 0.03) and oversensing of ventricular tachycardia/ventricular fibrillation (VT/VF) below the programmed rate zone (28.3%, *P* = 0.01) in year 1.

**Figure 4:**
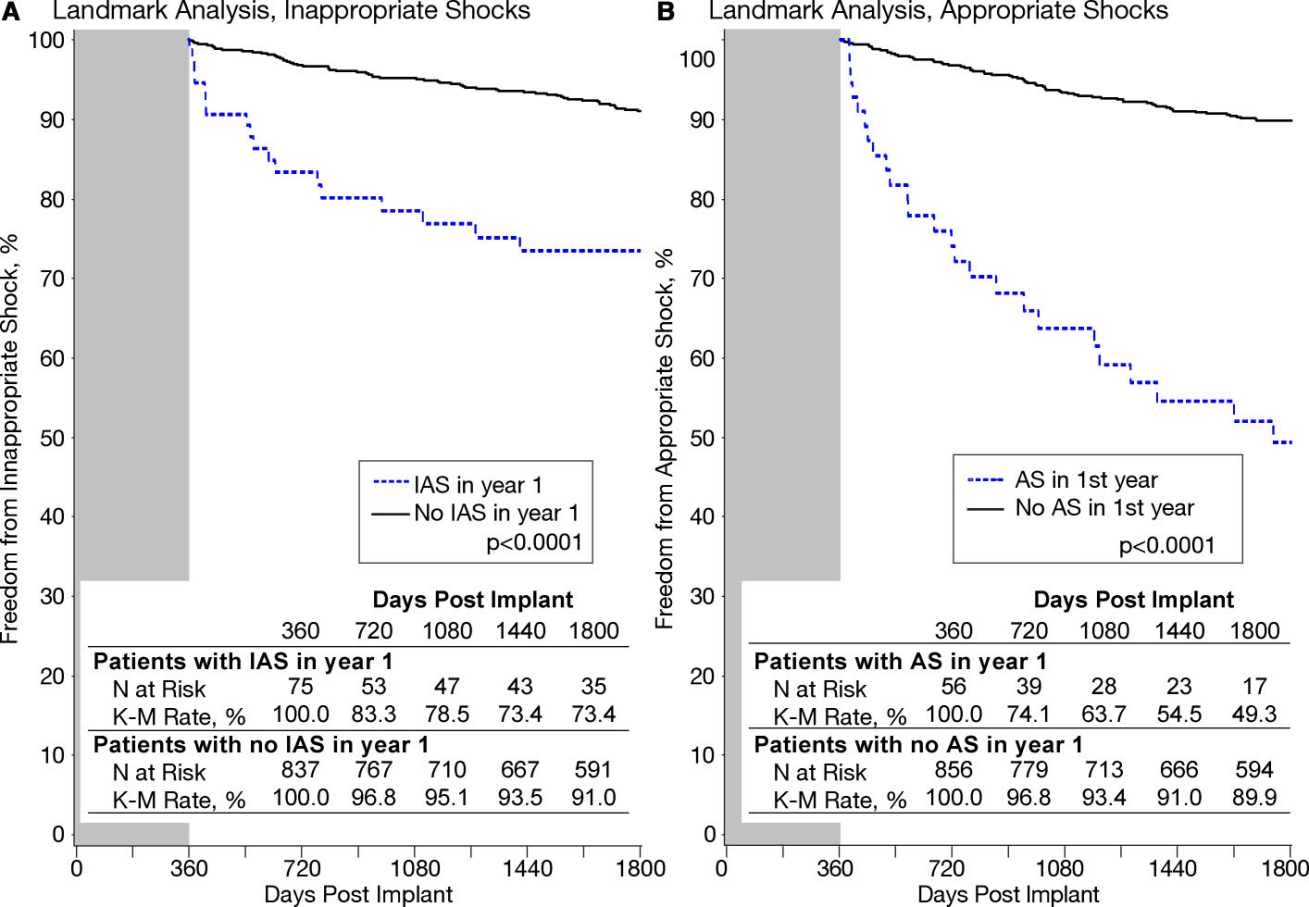
Kaplan–Meier event-free rates in in years 2–5, comparing patients who did and did not experience an event in year 1. (*A*) Inappropriate shocks. (*B*) Appropriate shocks. AS, appropriate shock; IAS, inappropriate shock.

Factors on univariable analysis that predicted one or more IAS in years 2–5 (Supplementary material online, *Table S5*) were AF, retrospective enrolment, implanted with a concomitant pacemaker, self-terminating inappropriate episodes in years 2–5, and IAS in year 1. Changes in programming in year 1 were not found to be a significant factor. The only multivariable predictor of one or more IAS in years 2–5 was self-terminating inappropriate episodes in years 2–5 [hazard ratio (HR) 4.7, 95% confidence interval (CI) 3.1–7.4, *P* < 0.0001]. Patients with IAS in year 1 had significantly more IAS in years 2–5 compared with patients who did not experience IAS in year 1 (*Figure 4A*).

Of the 83 patients with IAS in year 1, 57 (69%) had device reprogramming of the vector in 21 (25.3%), the therapy zone in 11 (13.3%), and both vector and therapy zone in 25 (30.1%). For the 36 patients with zone programming changes, mean programming changed (conditional, shock zone) from 200.0 ± 18.0 b.p.m. and 225.8 ± 14.8 b.p.m. to 217.5 ± 16.5 b.p.m. and 238.9 ± 11.2 b.p.m.

Following device reprogramming, these patients had a lower IAS rate in years 2–5, compared with patients with IAS in year 1 with no device reprogramming (Supplementary material online, *Figure S2*). The difference was not significant and rates converged after year 2.

### Appropriate shocks

**Figure 5:**
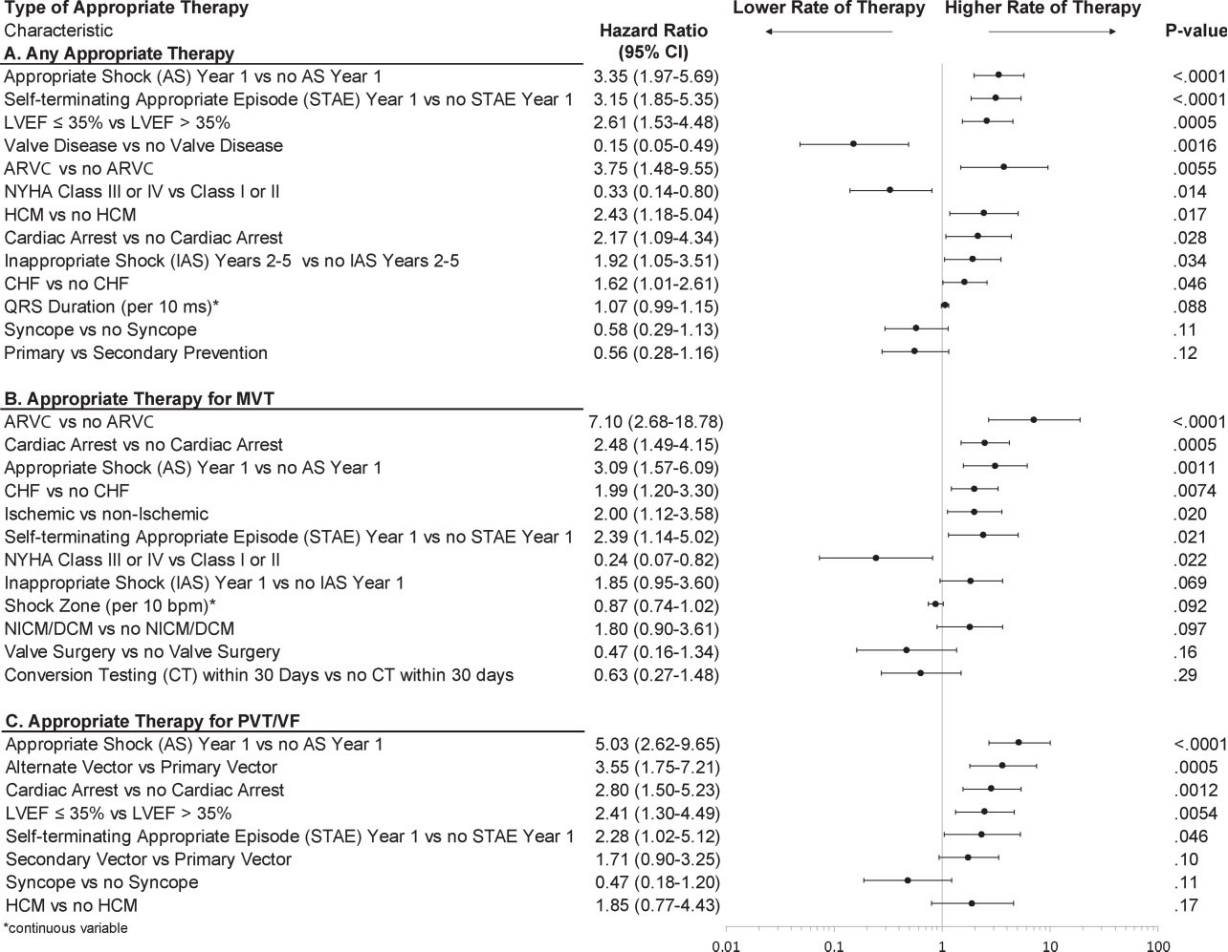
Significant multivariable predictors to the risk of receiving appropriate therapy using Anderson–Gill models. Separate models were constructed and are shown for any type of appropriate therapy (*A*), received and for receiving appropriate therapy for polymorphic ventricular tachycardia/ventricular fibrillation arrhythmias (*B*), and monomorphic ventricular tachycardia arrhythmias (*C*). Predictors modelled as continuous variables are indicated with an ‘asterisk’. ARVC, arrhythmogenic right ventricular cardiomyopathy; CHF, congestive heart failure; CI, confidence interval; DCM, dilated cardiomyopathy; HCM, hypertrophic cardiomyopathy; LVEF, left ventricular ejection fraction; NICM, non-ischaemic cardiomyopathy; NYHA, New York Heart Association.

AS therapies were delivered in 16% of patients in 5 years. Of the 62 patients (6.5%) receiving their first AS in year 1, 40.3% experienced further AS in years 2–5. Patients who experienced an AS in year 1 experienced significantly more AS in years 2–5 (*Figure 4B*) compared with patients with no AS in year 1. Supplementary material online, *Table S5* and *Figure 5A* shows univariable and multivariable predictors of one or more AS in years 2–5, respectively. The most significant predictors were prior AS and self-terminating ventricular arrhythmia episodes in year 1. Features of the severity of heart disease were predictors, such as LVEF, prior cardiac arrest, history of congestive heart failure, and LVEF ≤ 35%. New York Heart Association (NYHA) class I/II, ARVC, and HCM, and no valve disease were also significant predictors.

Overall, 146 patients experienced 310 discrete episodes of VT/VF: 86 patients with 161 monomorphic VT (MVT) episodes and 81 patients with 149 polymorphic VT/VF (PVT/VF) episodes. Thirty-seven of the 86 (43%) patients had >1 episode of MVT requiring shock therapy. Regarding self-terminating episodes, 44.6% of MVT and 21.2% of PVT/VF self-terminated without therapy being required.

In years 2–5, 62 (6.3%) patients had MVT and 67 (6.8%) patients had PVT/VF; 25 (21.7%) of the 115 patients had previous VT/VF in year 1.

Multivariable analysis identified several predictors of one or more treated episodes for MVT in years 2–5 including ARVC (HR 7.1), prior cardiac arrest, less severe heart failure in NYHA classes I–II, and ischaemic aetiology (*Figure 5B*). The main predictors of one or more PVT/VF episodes in years 2–5 (*Figure 5C*) included low LVEF (<35%), prior cardiac arrest, and AS in year 1. Self-terminating episodes in year 1 were significant predictors for both MVT and PVT/VF.

#### Long-term efficacy of shock therapy

**Figure 6:**
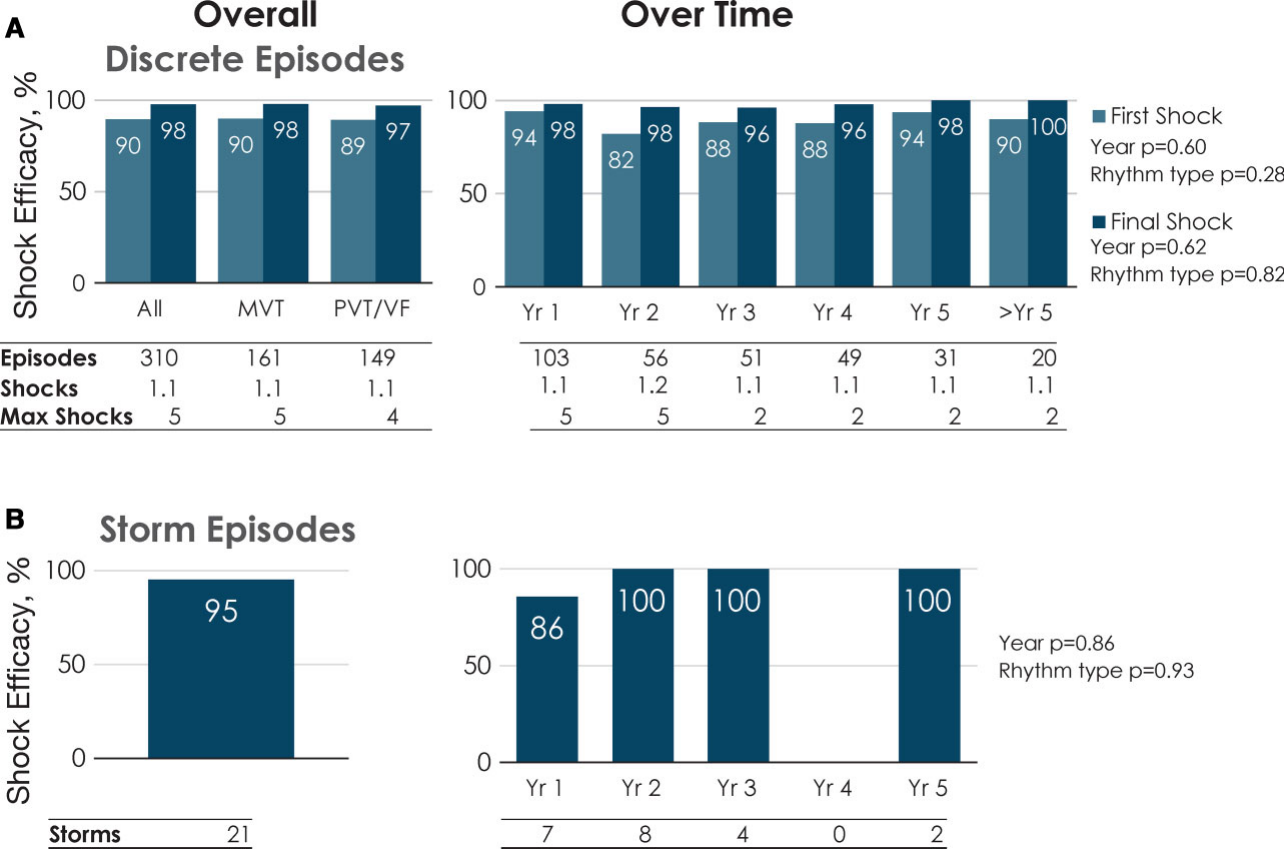
Shock efficacy of appropriate episodes experienced by patients in the EFFORTLESS study. (*A*) Discrete episodes. The left-sided histogram provides first- and final shock efficacy over the course of the study for all rhythms and for episodes with monomorphic ventricular tachycardia or polymorphic ventricular tachycardia/ventricular fibrillation rhythms. The right-sided histogram provides first- and final shock efficacy for episodes experienced for each post-implant year. The number of episodes, number of patients, mean number of shocks delivered, and maximum number of shocks delivered are listed below each rhythm type (left histogram) and follow-up year (right histogram). Regression analyses show that neither duration of follow-up (*P* = 0.60) nor rhythm type (*P* = 0.28) significantly impacted first shock efficacy nor final shock efficacy (*P* = 0.62 and *P* = 0.82, respectively) for discrete episodes. (*B*) Storm episodes. The left-sided histogram provides efficacy over the course of the study for all storm episodes. The right-sided histogram provides shock efficacy for storm episodes experienced for each post-implant year. The numbers of patients and storm episodes are provided for each data column. Regression analysis showed that that neither duration of follow-up (*P* = 0.86) nor rhythm type (*P* = 0.93) significantly impacted shock efficacy for storm episodes. MVT, monomorphic ventricular tachycardia; PVT, polymorphic ventricular tachycardia; VF, ventricular fibrillation; Yr, year.

The efficacy of shock therapy in cardioverting VT/VF was maintained over the 5-year period (*Figure 6A*, *Structured Graphical Abstract*) with an episode conversion rate of 98% (303 out of 310 episodes) and no significant effect of time since implant (*P* = 0.62). The 1st shock efficacy of 90% was similarly stable over time (*P* = 0.60). Rhythm type had no significant effect on conversion efficacy (*P* = 0.82) or first shock efficacy (*P* = 0.28). There were also no differences in efficacy per rhythm type or over time for storm episodes (*Figure 6B*, *Structured Graphical Abstract*). The final shock did not terminate the arrhythmias within the specified timeframe for success in seven patients; five of these patients were described previously^1^: in two patients, VT termination occurred after the fifth shock when the episode recording had finished. In another two patients, the device successfully terminated VF after additional shocks. One patient received multiple S-ICD and external shocks during a myectomy procedure and was successfully defibrillated externally.

One of the remaining two patients had a successful first shock for MVT but the arrhythmia (PVT) reinitiated and the subsequent shocks at maximal energy were unsuccessful although the VT self-terminated. A further episode reinitiated during which the arrhythmia was terminated by a shock delivered by the S-ICD. This patient had a diagnosis of ARVC and underwent an ablation as a result of experiencing these episodes. The second patient was experiencing cardiac arrest and had VF with multiple shocks and subsequently expired with the cause of the death determined to be ischaemic.

Out of the 21 storm episodes in 18 patients, 20 (95%) were successfully terminated with therapy; the one failed case of storm conversion was a patient with Loeffler’s cardiomyopathy with failed shocks but spontaneous conversion followed by severe bradycardia, as previously reported.^2^

The time to therapy was 17.6 s (IQR 15.8–20.6 s) and did not significantly change during the course of the study (Supplementary material online, *Figure S3*). Furthermore, there were no differences in time to therapy for MVT vs. PVT/VF. In seven patients, there were eight episodes where the time to therapy was >30 s. This was either because of MVT being near the rate cut-off threshold (*n* = 1), discrimination error (*n* = 1), or both (*n* = 1); or PVT/VF episode where undersensing (*n* = 3) signal noise (*n* = 1) or both (*n* = 1) were present. Time to therapy for storm episodes (17.3 s, IQR 15.2–20.2 s) also remained unchanged over time and was independent of rhythm type.

#### Generator replacement: defibrillation testing and device longevity

For the 71 patients with generator exchange, conversion testing was performed for 63 patients (88.7%) at original implant and for 34/71 (47.8%) at generator changeout. There are no reports of generator repositioning due to failed conversion testing during generator changeout.

At original implant, one patient was not inducible. Of the remaining 62 patients (83.7%), all 62 (100%) had conversion success at any energy and 58/62 (93.5%) were successfully converted at ≤65 J. At generator changeout, the 34 patients who underwent conversion testing had 100% conversion success at ≤65 J. The Fisher’s exact test *P*-values for comparing conversion success at original implant vs. generator changeout is 0.29 at ≤65 J and 1.00 at any energy.

Of these patients, 31 (43.7%) underwent conversion testing at both the initial and the replacement procedures. All 31 patients had successful conversion testing at both original implant and device replacement.

Device battery duration evaluated over a 5.1-year median follow-up, by design reflects only 42 patients whose longevity is shorter than their study follow-up, compared with the 597 patients whose original device had remaining battery life. A more accurate evaluation of battery longevity is anticipated in the EFFORTLESS sub-study (*n* = 200; NCT01085435, 8-year follow-up).

### Mortality

**Figure 7:**
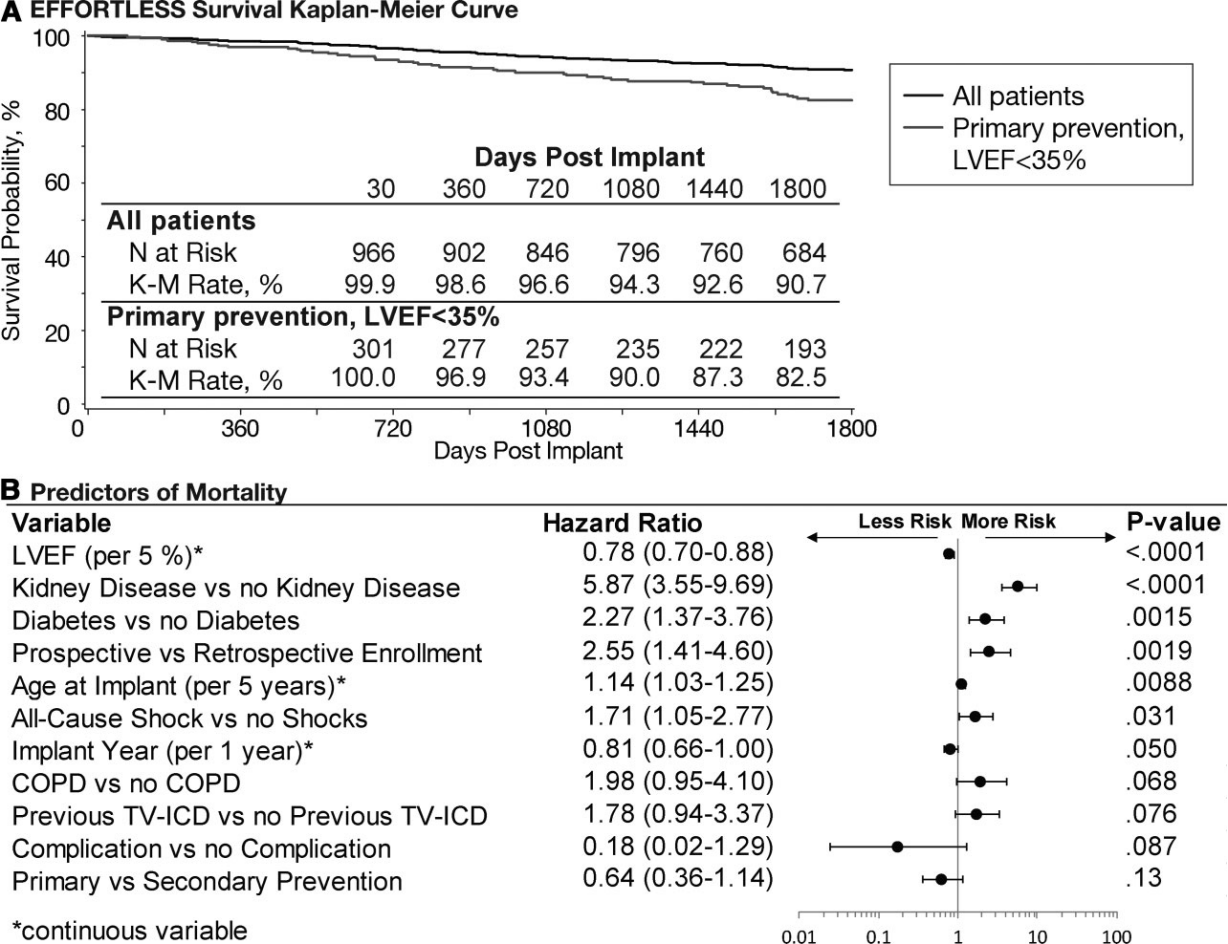
(*A*) Kaplan–Meir rate of survival for the EFFORTLESS study, 5-year follow-up, for all patients (*n* = 984) and for primary prevention, left ventricular ejection fraction (LVEF) ≤ 35% (*n* = 303). (*B*) Multivariable predictors of mortality. Predictors modelled as continuous variables are indicated with an ‘asterisk’. COPD, chronic obstructive pulmonary disease; LVEF, left ventricular ejection fraction; TV-ICD, transvenous implantable cardioverter-defibrillator.

After 5 years, 90.7% (95% CI 88.7–92.8%) of patients were still alive (*Figure 7A*). For primary prevention patients (*n* = 303), the 5-year survival rate was 82.5% for LVEF ≤ 35% compared with 99.5% for 202 LVEF > 35% patients. Supplementary material online, *Figure S4* illustrates the causes of death, the majority being mostly due to pump failure or non-cardiac causes. For seven patients, the cause of death was unknown. There were two arrhythmic deaths in addition to the previously reported arrhythmia storm case where the S-ICD had been inactivated in both: one patient with device inactivation for worsening heart failure and one patient following removal of life support. *Figure 7B* demonstrates the multivariable predictors of mortality, which included well-recognized prognostic factors such as lower LVEF, diabetes, age, and renal disease (note: univariable predictors of mortality are provided in Supplementary material online, *Table T5*). Prospective enrolment was a significant predictor on multivariable analysis; these patients were found previously to be significantly older with more comorbidities.^1^

## Discussion

This is the largest study to formally document the long-term outcomes of the S-ICD. The most important findings are: (i) shock efficacy of S-ICD therapy for prevention of sudden cardiac death was maintained over the 5-year period (*Structured Graphical Abstract*); (ii) only 2% of patients required S-ICD replacement for any kind of pacing indication; (iii) the majority of complications developed in the first few months after implant with a low attrition rate thereafter; (iv) only one suspected, unconfirmed, lead failure occurred (0.1%) during a median 5.1-year follow-up; and (v) treated and self-terminating arrhythmic episodes predicted future shock events, providing an opportunity for tailored optimization.

The longer-term follow-up, use of landmark analysis, and predictor models that account for multiple events have enabled evaluation of the long-term performance of the S-ICD. This provides a more robust evaluation of these determinants vs. prior early and mid-term EFFORTLESS publications,^1,2^ the recent UNTOUCHED trial,^5^ and randomized PRAETORIAN trial^6^; the latter two had shorter median follow-up of 1.5 and 4 years, respectively.

While analysing the findings of the EFFORTLESS registry, it must be noted that the EFFORTLESS study cohort is not the ‘typical’ ICD patient study population: patients are younger and have fewer comorbidities of heart failure, hypertension, diabetes, and kidney disease.

### Complications

The most common complications in EFFORTLESS included infection requiring replacement, the vast majority of which occurred in the first few months post-implant. Erosion occurred more commonly in years 2–5. Such complications were less frequent in the UNTOUCHED trial^5^ with 18-month infection rate of only 1.1 vs. 2.7% in 1 year in the US Post Approval Study (PAS)^7^ and 2.3% in 1 year in EFFORTLESS.^1^ This highlights an important evolution in experience and implantation techniques including the use of intermuscular placement reducing the risk of erosion. In comparison to TV-ICD infection risk, the PRAETORIAN trial,^6^ reported four (0.94%) infections in the S-ICD arm and eight (1.9%) infections in the TV-ICD arm over 4 years. While overall complication rates were not significantly different in PRAETORIAN, the only S-ICD vs. TV-ICD randomized trial, lead-related complications were significantly more common in the TV-ICD arm. Furthermore, there have been no reports of endocarditis types I–III complications in any S-ICD study.^5–8^ These reports support the hypothesis that S-ICD related complications are less severe and less costly than TV-ICD complications.

System performance was maintained over time with a limited number of failures, mainly due to premature battery depletion. Patients with prior history of valve surgery and cardiac arrest were more at risk for later complications whereas patients with a history of AF were of less risk, as were patients who underwent conversion testing within 30 days of device implant. Notably, neither experiencing complications in year 1, gender, nor body mass index (BMI) were multivariable predictors of later complications.

There was a single case of suspected lead defect-related noise requiring replacement (0.1%, annualized rate 0.02%), although subsequent investigation failed to identify a structural failure. This rate is lower than the reported 1.3% failure rate for contemporary leads^9^ and complies with the acceptable transvenous lead target failure rates of ≤0.4% annually.^10^ Subcutaneous implantable cardioverter-defibrillator leads have recently been reported to develop complications at annual rates of 0.22% for the newer model 3501 and 0.19% for Model 3010 and 3401 S-ICD leads, primarily related to lead fracture or sensing electrode failure.^11^ Replacement of the S-ICD lead was demonstrated to be complication-free in our study.

### Inappropriate shocks

The Kaplan–Meier IAS rate at 5 years increased by 5.2% from the 11.7% IAS rate reported for 3 years.^1^ Annualized IAS rate dropped to 2.1% in years 2–5 after an initial 8.7% in year 1. The main causes of IAS were cardiac oversensing, particularly T-wave oversensing (TWOS). The EFFORLTESS registry data illustrate the impact of improvements over time, in particular the SMART Pass filter,^12^ compared with the UNTOUCHED trial^5^ where the IAS rate was 3.1% in year 1. The drop in the annualized IAS rate indicates that once the S-ICD programming is optimized, there is a marked reduction in the risk of IASs due to TWOS and other causes. Notably, IAS due to AF or SVT in EFFORTLESS were present in only 2.9% patients (52 events) and just 0.6% patients (10 events) had discrimination errors.

The multivariable analysis highlights that sensed, self-terminating inappropriate episodes independently predicted one or more IAS in years 2–5. This provides an opportunity to review the patient and their device programming to prevent further IAS with reprogramming or treatment of AF/SVT if required. The availability of LATITUDE remote monitoring allows earlier intervention when such episodes are detected. In addition, there were relatively more IAS due to sources other than TWOS in years 2–5, particularly oversensing of VT/VF below the rate zone, due to double counting of the QRS complex or because of oversensed T-waves. Reprogramming after IAS experienced in year 1 did not lead to a significant difference in IAS rates in years 2–5, but the data were not powered for this analysis.

### Appropriate shocks

AS therapies were delivered in 16% of patients in 5 years, compared with 11.1% of patients as reported at mid-term.^1^ In this longer-term study, we were able to model a larger number of factors determining AS experienced in years 2–5 than previously reported including AS in year 1 and self-terminating episodes,^1,13^ both of which were highly significant. Patients having more than one episode of MVT (3.8%) may benefit from ATP or ablation/pharmaceutical intervention. Attempting to select for these patients, while weighing against the benefit of avoiding transvenous leads, is appropriate. Monomorphic VT was more common in ARVC and ischaemic heart disease patients, most likely indicative of fixed scar enabling stable re-entry as opposed to VF wavebreak. Hence, these data may help identify patients more likely to benefit from ATP or ablation.^14^ Multivariable modelling of ASs for MVT highlighted certain patient cohorts, specifically ARVC, as key predictors. However, the benefits of avoiding transvenous leads in these populations need to be weighed against the likelihood of MVT that could potentially be treated by ATP.

Interestingly, QRS duration was not a predictor of AS despite being a predictor of IAS. QRS duration has been a predictor of AS in TV-ICD trials but these trials focused on discrete populations of ischaemic and dilated cardiomyopathy as opposed to this more heterogeneous population.

### Self-terminating episodes

There were 233 self-terminating tachyarrhythmias, representing 34.4% of the sensed episodes in 13.2% of patients. The conditional zone with dual zone and delayed shock programming enable self-termination of non-sustained arrhythmias leading to reduced ASs and IASs.^15–19^ This reflects the findings of the recent PAS^7^ and UNTOUCHED^5^ data, which demonstrated the efficacy of this programming in avoiding unnecessary shocks that reduce the quality of life and survival. Previous TV-ICD studies report similar self-termination rates,^20–23^ including 34% in the shock-only arm of the Pacing Fast VT Reduces Shock Therapies (PainFREE Rx) II trial.^23^ In a previous analysis of primary and secondary prevention of S-ICD patients, 48% of the VT/VF episodes self-terminated without the need for treatment with a third of episodes occurring in secondary prevention cases.^13^

Reprogramming in year 1 for IAS reduced IAS to a degree, but this comes at a potential cost of under-detection of VT/VF. In 2.1% of patients, there were 53 episodes of VT/VF detected below the rate cut-off and more of these events took place later in the study. This phenomenon was also reported in PRAETORIAN in 11 (2.6%) patients, reported as appropriate therapy.^6^ Case studies have referred to these as ‘pseudoappropriate’^8^ and ‘inappropriately appropriate’,^9^ with the latter showing reduction of VT/VF oversensing with a SMART Pass software upgrade.

This raises the importance of monitoring for slower VT episodes that, if prolonged, may result in patient compromise. However, the experience of MADIT-RIT^19^ and the fact that such under-detection of slower VTs has not increased adverse outcomes provide some reassurance. Since self-terminating appropriate episodes were predictive of AS; remote LATITUDE monitoring may enable earlier preventative action, although more study is needed to guide appropriate intervention. Pre-emptive ablation in the BERLIN VT study failed to show a benefit in asymptomatic, untreated VT.^24^

### Shock efficacy

The ability to convert both storm and discrete events of VT/VF was consistent in each of the 5 years of follow-up. The first shock success for discrete episodes of 90% is equivalent to PAS^7^ and UNTOUCHED^5^ studies and compares favourably to TV-ICD trials such as SCD-HeFT (83%)^25^ and PainFREE Rx (87%).^23^ Multiple factors may conspire to reduce shock efficacy including myocardial ischaemia and acidosis.^26^ The low mortality rate from arrhythmic causes (0.3%) further demonstrates the effectiveness of the S-ICD shock therapy.

Recently, the maintenance of defibrillation efficacy was questioned due to 5 of 25 failures of DFT on generator replacement and proposing the possibility that fibrous capsule formation around the S-ICD generator increases shock impedance.^27^ This does not seem to be a common problem as shock efficacy was maintained throughout this follow-up and DFT was successful in 100% patients at generator change. It highlights the importance of ensuring optimal initial placement and securing of the device to deep fascia to avoid migration into a more anterior pocket, which will lower shock efficacy especially in high BMI patients.^28–30^

### Mortality

As with previous S-ICD studies, the annualized mortality rate is low at 1.86%. Although direct comparisons are not possible due to the heterogeneous nature of the cohort, the annual mortality rate of 3.5% in low LVEF primary prevention patients (*Figure 7*) is comparable to the rate of 3.2% in low LVEF, <55-year-old patients and compares favourably to 7.1% in 55–64-year olds reported in a meta-analysis of primary prevention ICD trials.^31^ There was one death in the primary prevention (annual mortality 0.1%) LVEF > 35% population.^32^

Well-recognized predictors of mortality were identified in the EFFORTLESS study: lower ejection fraction, diabetes, age, and renal disease. While all-cause shocks were a multivariable predictor of mortality, neither appropriate nor IASs alone was predictors as they were in the MADIT-RIT study of older, primary prevention patients.^33^ Notable missing predictors of mortality were ischaemic heart disease, the lack of DFT after device implant, and patients experiencing complications during the study.

Only 2/91 deaths were identified as arrhythmic deaths; for seven patients, the cause of death is unknown and could be arrhythmic in nature. Duray *et al*.^34^ show that predictors for arrhythmic mortality were NYHA class II [relative risk (RR) 5.4], NYHA class > II (11.8), VT as an indication for ICD therapy (RR 2.53) and amiodarone use (RR 3.95). As EFFORTLESS patients are much younger, have no pacing indications, and only 7.5% are NYHA class >II, a relatively low proportion of arrhythmic deaths compared with other studies with a more `typical’ ICD patient cohort^34–36^ is not surprising.

## Limitations

EFFORTLESS allowed for patient enrolment after device implant and as such rates of mortality may be affected by inclusion bias as previously described.^1^ The registry records outcomes in a high proportion of Gen 1 devices before the advent of SMART Pass filtering so the full impact of this could not be assessed in this study. Complications and IAS rates reflect the learning curve of implanting a device early in its global experience.^37^ The impact of generator changes on infection requiring extraction will require longer-term evaluation.

## Conclusions

The EFFORTLESS registry is the largest and longest follow-up study of S-ICD efficacy and patient outcomes. A high level of VT/VF shock efficacy was maintained over the median 5.1-year follow-up, along with a low complication rate, a very low percentage of conversion to transvenous devices, and few IASs for AF/SVT. Treated and self-terminating arrhythmic episodes predict future shock events in the long-term patient experience, while complications in the first year are not significant in the prognosis of late complications. Future studies including EFFORTLESS sub-study (*n* = 200; NCT01085435, 8-year follow-up) will focus on the impact of generator changes, providing data for much longer-term comparisons with TV-ICDs when the impact of chronically implanted lead issues will become more evident.

## Supplementary Material

ehab921_Supplementary_Data
