# Supplementary material for: Subcutaneous implantable cardioverter-defibrillators: long-term results of the EFFORTLESS study
Source: Eur Heart J. 2022 Jan 28;43(21):2037–50. doi: 10.1093/eurheartj/ehab921 (PMC9156377; doi:10.1093/eurheartj/ehab921)

Supplementary Methods, Tables, and Figures

Supplementary Methods

Significance in time to first event was evaluated using a log rank test. Multivariable analyses using the Anderson-Gill model, which factors in predictors for multiple events, were performed separately for the year 2-5 outcomes of complications, appropriate therapy, therapy for polymorphic ventricular tachycardia (PVT) or ventricular fibrillation (VF), therapy for monomorphic ventricular tachycardia (MVT), therapy, and IAS. Events that took place in year 1 were included as predictors for events in years 2-5. The Anderson-Gill model incorporates a robust sandwich variance estimator to correct for the correlation between recurring events within the same subject. Multivariable analysis using the Cox proportional hazards model was performed for mortality. Univariable analysis for each model was performed for inclusion in multivariable modeling. Backward selection (α=20%) was used to determine the final multivariable model. Effects of time and rhythm type on first and final shock efficacy were evaluated using logistic regression and effects of time and rhythmtype on time to therapy were evaluated using a linear regression preceded by logarithmic transformation.

Supplementary Table 1 Patient Characteristics

Parameter	Results (n=984)
Age at implantation, years	48.4 ± 17
Female Gender	275 (27.9)
BMI, kg/m^2	27.1 ± 5.7
Ejection fraction, %	43.4 ± 18.2
QRS duration, ms	105.9 ± 24.6
NYHA Class	
None	715 (72.7)
I/II	170 (17.3)
III/IV	72 (7.3)
Unknown	27 (2.7)
Primary Prevention	593 (65.2)
Ejection fraction < 35%	303 (30.8)
Ischemic etiology	288 (32.4)
**Comorbidities**	
Hypertension	279 (28.4)
MI	277 (28.2)
Cardiac arrest	275 (27.9)
Congestive heart failure	269 (27.3)
Syncope	181 (18.4)
AF	158 (16.1)
Valve disease	123 (12.5)
Diabetes	111 (11.3)
Kidney disease	82 (8.3)
Stroke (including TIA)	52 (5.3)
COPD	51 (5.2)
**Cardiac Surgery**	
Previous transvenous ICD	139 (14.1)
Due to infection	73 (7.4)
CABG	78 (7.9)
Valve surgery	62 (6.3)
Pacemaker (PM) implant	30 (3.0)
Previous PM, explanted	5 (0.5)
Concomitant PM	25 (2.5)
**Primary cardiac disease**	
Previous MI/Ischemia/CAD	282 (28.6)
NICM/DCM	175 (17.8)
Hypertrophic cardiomyopathy	106 (10.8)
Channelopathy	199 (20.2)
Genetic	49 (5.0)
ARVD	32 (3.2)
Valvular disease	21 (2.1)
Other	45 (4.6)
Unknown	76 (7.7)

Values are mean ± SD or n (%). All values are simple percentages

AF = atrial fibrillation; BMI = body mass index; CABG = coronary artery bypass graft; CAD = coronary artery disease; COPD = chronic obstructive pulmonary disease; ICD = implantable cardioverter-defibrillator; K-M = Kaplan-Meier; MI = myocardial infarction; TIA = transient ischemic attack.

Supplementary Table 2: Reasons for Study Exit

**Reason for Study Exit**	**Number of patients (%)**
Deceased	91 (9.2)
Explanted	87 (8.8)
Infection	*25 (2.5)*
Change in Indication	*19 (1.9)*
Inappropriate Shocks	*12 (1.2)*
Erosion	*9 (0.9)*
Discomfort	*6 (0.6)*
Change in Patient Condition	*6 (0.6)*
Failure to convert during induction testing	*4 (0.4)*
Patient Request/Elective Decision	*4 (0.4)*
Lack of Patient Participation	*1 (0.1)*
Investigator Discretion	*1 (0.1)*
Withdrawal	84 (8.5)
Lack of Patient Participation	*64 (6.5)*
Change in Indication	*8 (0.8)*
Inclusion/Exclusion Deviation	*1 (0.1)*
Investigator Discretion	*4 (0.4)*
Change in Patient Condition	*3 (0.3)*
Patient Request/Elective Decision	*3 (0.3)*
Inappropriate Shocks	*1 (0.1)*
Completed Initial Study per Protocol	703 (71.4)
Site Terminated	19 (1.9)
**Total**	**984 (100.0)**

Supplementary Table 3a: Summary of Pulse Generator Replacements

Reason for PG replacement	# Patients
**ERI**	34
**Infection requiring device removal**	8
**Premature Cell Battery Depletion**	8
**Erosion**	6
**Discomfort**	4
**Other technical complications**	3
**Inability to Communicate with the Device**	2
**PG Movement**	2
**Electrode Movement**	1
**Inappropriate Shock Requiring Intervention: Cardiac Oversensing**	1
**Inappropriate Shock Requiring Intervention: Non-Cardiac Oversensing**	1
**Infection requiring device removal & Erosion**	1
**Total**	**71**

Supplementary Table 3b: Summary of Electrode Replacements

Reason for Electrode Replacement	Notes	# Patients
Infection requiring device removal	In at least 7 cases, complete extraction, wait for wound healing, and then re-implant with new system.<br><br>In at least 2 cases, infection location at electrode	9
Other Procedural Complications	Electrode damaged during procedure	2
Discomfort	Discomfort at electrode	1
Electrode Movement		1
Inappropriate Shock Requiring Intervention: Non-Cardiac Oversensing	Noise source suspected to be due to lead defect*	1
ERI	Conservative extraction of entire system at normal ERI	1
**Total**		**15**

* 3.5 years after implant procedure. Root cause of noise source remains unknown; lead defect not identified.

Supplementary Table 4: Complications by Gender and Time Period

Complication Term	Total		Male				Female			
			Year 1		> Year 1		Year 1		> Year 1	
	Events	Subjects N (%)	Events	Subjects N (%)		Subjects N (%)	Events	Subjects N (%)	Events	Subjects N (%)
Infection requiring device removal	35	32 (3.3)	21	20 (2.8)	7	7 (1.0)	5	5 (1.8)	2	2 (0.7)
Erosion	24	24 (2.4)	8	8 (1.1)	10	10 (1.4)	2	2 (0.7)	4	4 (1.5)
Inappropriate Shock: Cardiac Oversensing	17	16 (1.6)	4	4 (0.6)	9	8 (1.1)	1	1 (0.4)	3	3 (1.1)
Other procedural complications	13	13 (1.3)	6	6 (0.8)	2	2 (0.3)	1	1 (0.4)	4	4 (1.5)
Discomfort	11	11 (1.1)	1	1 (0.1)	0	0 (0.0)	2	2 (0.7)	8	8 (2.9)
Hematoma	9	9 (0.9)	6	6 (0.8)	1	1 (0.1)	2	2 (0.7)	0	0 (0.0)
PG Movement	8	6 (0.6)	5	3 (0.4)	2	2 (0.3)	0	0 (0.0)	1	1 (0.4)
Premature Cell Battery Depletion	8	8 (0.8)	1	1 (0.1)	4	4 (0.6)	1	1 (0.4)	2	2 (0.7)
Sub-optimal Electrode position	8	8 (0.8)	5	5 (0.7)	0	0 (0.0)	3	3 (1.1)	0	0 (0.0)
Electrode Movement	7	7 (0.7)	3	3 (0.4)	0	0 (0.0)	3	3 (1.1)	1	1 (0.4)
Incision/Superficial Infection	6	6 (0.6)	2	2 (0.3)	1	1 (0.1)	3	3 (1.1)	0	0 (0.0)
Unable to Convert: During Procedure	5	5 (0.5)	4	4 (0.6)	1	1 (0.1)	0	0 (0.0)	0	0 (0.0)
Inappropriate Shock: Non-Cardiac Oversensing	4	4 (0.4)	1	1 (0.1)	0	0 (0.0)	1	1 (0.4)	2	2 (0.7)
Inappropriate Shock: SVT Above Discrimination Zone (Normal Device Function)	4	4 (0.4)	1	1 (0.1)	2	2 (0.3)	0	0 (0.0)	1	1 (0.4)
Other technical complications	4	4 (0.4)	1	1 (0.1)	1	1 (0.1)	0	0 (0.0)	2	2 (0.7)
Inability to Communicate with the Device	3	3 (0.3)	0	0 (0.0)	2	2 (0.3)	0	0 (0.0)	1	1 (0.4)
Sub-optimal PG and Electrode position	3	3 (0.3)	3	3 (0.4)	0	0 (0.0)	0	0 (0.0)	0	0 (0.0)
Sub-optimal PG position	1	1 (0.1)	0	0 (0.0)	0	0 (0.0)	1	1 (0.4)	0	0 (0.0)

PG = Pulse generator; SVT= supraventricular tachycardia.

Supplementary Table 5: Univariable Models

	Appropriately Treated Episodes Years 2-5		Treated PVT/VF Episodes, Years 2-5		Treated MVT Episodes, Years 2-5		Inappropriately Treated Episodes, Years 2-5		Complications, Years 2-5		Mortality	
**Predictor**	**HR (95% CI)**	**P-value**	**HR (95% CI)**	**P-value**	**HR (95% CI)**	**P-value**	**HR (95% CI)**	**P-value**	**HR (95% CI)**	**P-value**	**HR (95% CI)**	**P-value**
AF vs no AF	1.259 (0.863, 1.837)	0.2321	1.256 (0.735, 2.146)	0.4035	1.687 (1.021, 2.786)	0.0412	1.599 (1.031, 2.478)	0.0359	**1.054 (0.526, 2.113)**	**0.8824**	3.128 (1.964, 4.981)	<.0001
Age at Implant (per 5 years)*	0.995 (0.952, 1.041)	0.8367	0.996 (0.930, 1.068)	0.9185	1.021 (0.943, 1.105)	0.6115	1.032 (0.981, 1.086)	0.2212	1.013 (0.944, 1.087)	0.7239	**1.425 (1.31, 1.551)**	**<.0001**
Female vs Male	0.826 (0.585, 1.166)	0.2776	0.901 (0.579, 1.403)	0.6437	0.54 (0.317, 0.92)	0.0236	0.866 (0.582, 1.288)	0.4776	1.868 (1.164, 2.998)	0.0097	0.606 (0.345, 1.063)	0.0807
BMI (per 1 kg/m²)*	1.026 (0.998, 1.054)	0.068	1.025 (0.986, 1.066)	0.2189	1.033 (1.003, 1.065)	0.0315	1.004 (0.976, 1.033)	0.7829	1.039 (0.991, 1.09)	0.1165	1.031 (0.992, 1.072)	0.1164
Ischemic vs non-Ischemic	1.406 (1.031, 1.916)	0.0312	1.463 (0.938, 2.283)	0.0936	**1.514 (0.986, 2.324)**	**0.0578**	1.098 (0.742, 1.623)	0.6407	0.811 (0.488, 1.348)	0.4189	4.955 (3.059, 8.027)	<.0001
LVEF (per 5 %)*	0.948 (0.901, 0.997)	0.0373	0.954 (0.890, 1.023)	0.1842	0.938 (0.87, 1.012)	0.0974	1.011 (0.958, 1.066)	0.6979	0.985 (0.898, 1.08)	0.7472	**0.746 (0.684, 0.813)**	**<.0001**
LVEF ≤ 35% vs LVEF > 35%	**1.775 (1.244, 2.533)**	**0.0016**	**1.805 (1.088, 2.995)**	**0.0222**	1.728 (1.097, 2.721)	0.0182	0.862 (0.566, 1.315)	0.4916	1.171 (0.64, 2.146)	0.6085	6.091 (3.341, 11.106)	<.0001
QRS Duration (per 10 ms)*	1.051 (0.989, 1.117)	0.1113	1.057 (0.965, 1.158)	0.2326	1.043 (0.958, 1.136)	0.334	1.06 (0.993, 1.131)	0.0827	1.102 (1.015, 1.197)	0.0211	1.201 (1.121, 1.288)	<.0001
NYHA Class III or IV vs Class I or II	**0.635 (0.339, 1.190)**	**0.1564**	1.176 (0. 411, 3.363)	0.7618	**0.575 (0.298, 1.11)**	**0.0991**	1.22 (0.608, 2.449)	0.5763	0.966 (0.303, 3.083)	0.9538	8.065 (4.938, 13.173)	<.0001
Diabetes vs no Diabetes	1.097 (0.696, 1.728)	0.6913	0.716 (0.286, 1.795)	0.4766	1.65 (0.991, 2.748)	0.0543	0.736 (0.337, 1.609)	0.4428	0.621 (0.277, 1.396)	0.2492	**5.575 (3.537, 8.787)**	**<.0001**
Kidney Disease vs no Kidney Disease	1.193 (0.697, 2.042)	0.205	1.556 (0.810, 2.988)	0.1840	1.201 (0.576, 2.503)	0.6247	0.962 (0.382, 2.422)	0.9353	0.769 (0.242, 2.446)	0.6569	**12.643 (8.098, 19.74)**	**<.0001**
Primary vs Secondary Prevention	0.632 (0.460, 0.868)	0.0046	0.382 (0.235, 0.623)	0.0001	0.777 (0.503, 1.201)	0.2559	1.032 (0.709, 1.503)	0.8676	0.685 (0.415, 1.132)	0.14	1.047 (0.655, 1.674)	0.8478
Concomitant Pacemaker vs no Concomitant Pacemaker	0.683 (0.150, 3.115)	0.6224	0.798 (0.118, 5.419)	0.8177	0.753 (0.2, 2.826)	0.6738	3.537 (1.686, 7.42)	0.0008	2.614 (0.88, 7.766)	0.0837	5.547 (2.668, 11.535)	<.0001
Previous ICD vs no Previous ICD	1.193 (0.792, 1.797)	0.3985	1.643 (1.006, 2.684)	0.0475	1.006 (0.563, 1.798)	0.984	0.79 (0.442, 1.414)	0.4278	1.1 (0.597, 2.027)	0.7593	1.794 (1.049, 3.068)	0.0328
Channelopathy vs no Channelopathy	0.798 (0.530, 1.203)	0.2823	1.279 (0.820, 1.996)	0.2785	0.14 (0.034, 0.573)	0.0063	0.674 (0.421, 1.077)	0.099	1.454 (0.834, 2.537)	0.1871	0.097 (0.024, 0.396)	0.0011
HCM vs no HCM	**0.757 (0.479, 1.197)**	**0.2336**	0.709 (0.375, 1.343)	0.2916	0.605 (0.22, 1.662)	0.3292	1.446 (0.874, 2.392)	0.1511	0.71 (0.291, 1.735)	0.4523	N/A	0.9806
NICM/DCM vs no NICM/DCM	0.950 (0.636, 1.420)	0.8029	0.878 (0.490, 1.575)	0.6629	0.916 (0.552, 1.52)	0.7348	1.365 (0.927, 2.009)	0.1152	1.204 (0.668, 2.17)	0.5378	1.013 (0.568, 1.804)	0.9656
Genetic vs no Genetic	0.759 (0.336, 1.717)	0.5079	0.296 (0.042, 2.085)	0.2219	0.755 (0.319, 1.787)	0.5227	1.071 (0.547, 2.097)	0.8408	0.818 (0.355, 1.889)	0.6388	0.461 (0.113, 1.878)	0.28
Brugada vs no Brugada	0.297 (0.095, 0.927)	0.0366	0.525 (0.166, 1.661)	0.2731	N/A	N/A	0.67 (0.354, 1.266)	0.2171	1.79 (0.904, 3.548)	0.0951	N/A	0.9827
Conversion Testing(CT) within 30 Days vs no CT within 30days	0.877 (0.458, 1.680)	0.6918	1.486 (0.377, 5.86)	0.5718	0.588 (0.296, 1.167)	0.1291	0.889 (0.477, 1.655)	0.7106	**0.552 (0.232, 1.316)**	**0.1800**	0.506 (0.233, 1.1)	0.0855
Antiarrhythmic vs no Antiarrhythmic	1.462 (0.864, 2.475)	0.1570	0.682 (0.22, 2.109)	0.5062	2.078 (1.244, 3.47)	0.0052	1.194 (0.53, 2.69)	0.6693	0.943 (0.346, 2.569)	0.9089	1.726 (0.862, 3.455)	0.1234
CABG vs no CABG	1.156 (0.633, 2.109)	0.6378	0.67 (0.215, 2.091)	0.4903	1.463 (0.703, 3.042)	0.3087	1.59 (0.848, 2.98)	0.1481	1.739 (0.803, 3.765)	0.1602	3.681 (2.152, 6.299)	<.0001
Cardiac Arrest vs no Cardiac Arrest	**1.770 (1.273, 2.461)**	**0.0007**	**2.67 (1.545, 4.612)**	**0.0004**	**1.52 (0.983, 2.35)**	**0.0594**	0.919 (0.611, 1.382)	0.6846	**1.717 (1.036, 2.844)**	**0.0358**	0.834 (0.498, 1.397)	0.4897

	Appropriately Treated Episodes Years 2-5		Treated PVT/VF Episodes, Years 2-5		Treated MVT Episodes, Years 2-5		Inappropriately Treated Episodes, Years 2-5		Complications, Years 2-5		Mortality	
**Predictor**	**HR (95% CI)**	**P-value**	**HR (95% CI)**	**P-value**	**HR (95% CI)**	**P-value**	**HR (95% CI)**	**P-value**	**HR (95% CI)**	**P-value**	**HR (95% CI)**	**P-value**
CHF vs no CHF	**1.290 (0.921, 1.808)**	**0.1382**	1.244 (0.763, 2.028)	0.3806	**1.494 (0.959, 2.327)**	**0.0758**	0.772 (0.512, 1.165)	0.2178	0.777 (0.448, 1.348)	0.3694	6.251 (3.909, 9.997)	<.0001
COPD vs no COPD	0.980 (0.470, 2.044)	0.9564	0.769 (0.196, 3.017)	0.7066	1.134 (0.474, 2.709)	0.7778	1.478 (0.681, 3.205)	0.323	1.198 (0.385, 3.72)	0.7553	3.654 (1.932, 6.91)	<.0001
Hypertension vs no Hypertension	0.916 (0.640, 1.312)	0.6337	0.931 (0.551, 1.575)	0.7908	1.024 (0.662, 1.583)	0.9157	1.03 (0.586, 1.81)	0.919	0.94 (0.56, 1.578)	0.8137	3.288 (2.112, 5.12)	<.0001
Stroke vs no Stroke	1.270 (0.624, 2.585)	0.5096	0.998 (0.351, 2.838)	0.9973	1.551 (0.768, 3.132)	0.2213	0.67 (0.167, 2.691)	0.5724	0.666 (0.16, 2.773)	0.5765	1.908 (0.878, 4.146)	0.1026
Syncope vs no Syncope	0.810 (0.534, 1.230)	0.3235	0.64 (0.352, 1.163)	0.143	0.774 (0.428, 1.403)	0.399	1.183 (0.757, 1.851)	0.4607	1.283 (0.749, 2.198)	0.3635	0.661 (0.341, 1.283)	0.2211
Valve Disease vs no Valve Disease	**1.125 (0.698, 1.813)**	**0.6289**	1.349 (0.695, 2.618)	0.3757	1.231 (0.739, 2.052)	0.4251	1.538 (0.94, 2.515)	0.0865	1.573 (0.812, 3.05)	0.1795	3.427 (2.121, 5.537)	<.0001
Valve Surgery vs no Valve Surgery	0.745 (0.380, 1.460)	0.3914	1.116 (0.401, 3.111)	0.8333	0.711 (0.366, 1.381)	0.3146	1.63 (0.85, 3.128)	0.1417	**2.733 (1.343, 5.561)**	**0.0055**	2.552 (1.315, 4.955)	0.0056
Previous MI/Ischemia/CAD vs no Previous MI/Ischemia/CAD	1.379 (1.007, 1.888)	0.0454	1.365 (0.862, 2.162)	0.1847	1.421 (0.933, 2.165)	0.1014	0.954 (0.62, 1.467)	0.829	0.752 (0.44, 1.286)	0.2978	3.763 (2.406, 5.886)	<.0001
ARVD vs no ARVD	**1.701 (0.941, 3.073)**	**0.0785**	0.505 (0.071, 3.585)	0.4945	**3.692 (1.808, 7.537)**	**0.0003**	1.084 (0.359, 3.276)	0.8859	1.593 (0.509, 4.99)	0.4242	N/A	0.9839
Prospective vs Retrospective Enrollment	1.256 (0.936, 1.686)	0.1284	1.103 (0.731, 1.664)	0.6396	1.317 (0.877, 1.976)	0.1842	0.708 (0.505, 0.994)	0.046	0.813 (0.492, 1.345)	0.4203	**2.792 (1.718, 4.536)**	**<.0001**
Implant Year (per 1 year)*	1.035 (0.951, 1.126)	0.4262	1.07 (0.942, 1.216)	0.2959	0.995 (0.89, 1.112)	0.9273	0.957 (0.844, 1.086)	0.4967	0.948 (0.8, 1.124)	0.5374	**1.01 (0.866, 1.178)**	**0.8985**
Inappropriate Shock (IAS) Year 1 vs no IAS Year 1	1.199 (0.769, 1.868)	0.4232	1.092 (0.445, 2.684)	0.8472	1.258 (0.733, 2.16)	0.4046	1.978 (1.169, 3.349)	0.011	0.949 (0.435, 2.067)	0.8946		
Appropriate Shock (AS) Year 1 vs no AS Year 1	**2.727 (1.713, 4.340)**	**<.0001**	**4.006 (2.126, 7.547)**	**<.0001**	**3.504 (1.99, 6.168)**	**<.0001**	1.214 (0.579, 2.544)	0.6076	0.795 (0.338, 1.871)	0.5997		
Self-terminating Appropriate Episode (STAE) Year 1 vs no STAE Year 1	**2.153 (1.361, 3.407)**	**0.0011**	**3.358 (1.927, 5.852)**	**<.0001**	**2.986 (1.587, 5.618)**	**0.0007**						
Self-terminating Inappropriate Episode (STIE) Year 1 vs no STIE Year 1							1.572 (0.947, 2.61)	0.0802				
Complication Year 1 vs no Complication Year 1									1.348 (0.615, 2.956)	0.4555		
Inappropriate Shoc (IAS) Years 2-5 vs no IAS Years 2-5	**1.127 (0.714, 1.780)**	**0.6075**	1.277 (0.536, 3.046)	0.5811	1.09 (0.645, 1.84)	0.748						
Appropriate Shock (AS) Years 2-5 vs no AS Years 2-5							1.699 (0.843, 3.424)	0.1382	1.117 (0.584, 2.136)	0.739		
Self-terminating Inappropriate Episode (STIAE) Years 2-5 vs no STIAE Years 2-5							**4.775 (2.605, 8.75)**	**<.0001**				
Self-terminating Appropriate Episode (STAE) Years 2-5 vs no STAE Years 2-5	0.867 (0.560, 1.341)	0.5202	1.111 (0.595, 2.074)	0.7408	0.754 (0.438, 1.296)	0.3064						
Inappropriate Shock (IAS) vs no IAS											1.52 (0.889, 2.599)	0.1264
Appropriate Shock (AS) vs no AS											2.128 (1.301, 3.481)	0.0026
All-Cause Shock vs no Shocks											**1.902 (1.216, 2.975)**	**0.0048**
Complication vs no Complication											0.316 (0.1, 1.001)	0.0501

	Appropriately Treated Episodes Years 2-5		Treated PVT/VF Episodes, Years 2-5		Treated MVT Episodes, Years 2-5		Inappropriately Treated Episodes, Years 2-5		Complications, Years 2-5		Mortality	
**Predictor**	**HR (95% CI)**	**P-value**	**HR (95% CI)**	**P-value**	**HR (95% CI)**	**P-value**	**HR (95% CI)**	**P-value**	**HR (95% CI)**	**P-value**	**HR (95% CI)**	**P-value**
PG Changeout vs no PG Changeout											0.171 (0.024, 1.229)	0.0793
Gen2 PG (A209) vs Gen 1 PG (1010)	2.281 (0.850, 6.121)	0.1014	2.53 (0.821, 7.8)	0.1062	N/A	N/A	1.093 (0.139, 8.587)	0.9328	1.097 (0.246, 4.9)	0.9036		
Gen3 PG (A219) vs Gen 1 PG (1010)	N/A	N/A	N/A	N/A	N/A	N/A	4.013 (0.579, 27.826)	0.1596	N/A	N/A		
Shock Zone (per 10 bpm)*	0. 990 (0.886, 1.108)	0.8648	1.05 (0.893, 1.235)	0.5522	0.884 (0.773, 1.012)	0.0744	0.903 (0.786, 1.036)	0.1458				
Conditional Zone (per 10 bpm)*	0.993 (0.913, 1.079)	0.8635	1.029 (0.92, 1.15)	0.6191	0.922 (0.806, 1.055)	0.2382	0.954 (0.846, 1.075)	0.4421				
Gain x2 vs Gain x1	0.874 (0.489, 1.562)	0.6486	1.298 (0.574, 2.933)	0.5306	0.95 (0.441, 2.045)	0.8953	1.048 (0.573, 1.918)	0.8796				
Alternate Vector vs Primary Vector	1.308 (0.842, 2.032)	0.2327	**2.255 (1.314, 3.872)**	**0.0032**	0.895 (0.369, 2.168)	0.8054	1.043 (0.522, 2.085)	0.9042				
Secondary Vector vs Primary Vector	1.049 (0.750, 1.467)	0.7819	1.451 (0.889, 2.37)	0.1366	0.852 (0.551, 1.316)	0.4697	1.235 (0.796, 1.916)	0.3454				
Discrimination Zone Width (per 10 units)	1.000 (0.924, 1.083)	0.9920	1.002 (0.901, 1.115)	0.9665	0.976 (0.85, 1.12)	0.7276	0.976 (0.878, 1.086)	0.6566				
200/250 Programming vs Other Programming	1.190 (0.700, 2.022)	0.5211	1.023 (0.478, 2.189)	0.9538	1.237 (0.609, 2.512)	0.5569	0.529 (0.256, 1.097)	0.0871				
Change in Shock Zone - Year 1 vs no Change	0.700 (0.444, 1.104)	0.1254	0.583 (0.24, 1.42)	0.2352	0.908 (0.546, 1.511)	0.7107	1.428 (0.832, 2.451)	0.1966				
Change in Conditional Zone - Year 1 vs no Change	0.665 (0.437, 1.011)	0.0562	0.976 (0.453, 2.103)	0.9500	0.767 (0.462, 1.273)	0.3055	1.525 (0.877, 2.651)	0.1352				
Change in Vector - Year 1 vs no Change	1.380 (1.003, 1.899)	0.0482	1.686 (1.131, 2.512)	0.0103	1.152 (0.712, 1.864)	0.5648	0.929 (0.577, 1.496)	0.7625				
Change in Gain - Year 1 vs no Gain	0.470 (0.210, 1.049)	0.0652	0.214 (0.029, 1.562)	0.1284	0.631 (0.278, 1.43)	0.2696	1.257 (0.626, 2.524)	0.5208				
Any Change in Programming - Year 1 vs no Change	1.461 (0.985, 2.168)	0.0594	1.253 (0.625, 2.511)	0.5247	3.51 (0.496, 24.852)	0.2086	1.461 (0.985, 2.168)	0.0594				

Dark grey cells: not included in predictor model

Bolded text: significant multivariable predictors

Supplementary Table 6: Study Sites and Investigators

Investigational Site	Primary Investigator
Aalborg Univeristiy Hospital	Søren Hjortshǿj
Aarhus University Hospital, Skejby	Jens Nielsen
Amsterdam University Medical Centers	Reinoud Knops
Arnas Garibaldi Nesima	Michele Gulizia
Auckland City Hospital	Margaret Hood
Azienda Ospedaliero Universitaria Padova	Emanuele Bertaglia
Azienda Ospedaliero Universitaria Pisana	Maria Grazia Bongiorni
Barts Heart Centre	Pier Lambiase
Bristol Royal Hospital	Graham Stuart
Catharina Hospital	Lucas Dekker
CHU La Timone	Jean-Claude Deharo
Complexo Hospitalario Universitario A Coruna	Luisa Pérez
Erasmus Medical Centre	Dominic Theuns
Hôpital Cardiologique du Haut-L'évêque	Pierre Bordachar
Hospital Santa Cruz	Pedro Adragão
Immanuel Klinikum Bernau	Christian Butter
John Radcliffe Hospital	Tim Betts
Kings College Hospital	Frances Murgatroyd
Klinikum Bielefeld	Christoph Stellbrink
Klinikum Coburg	Johannes Brachmann
Maastricht UMC	Kevin Vernooy
Marienkrankenhaus Papenburg	Andreas Wilke
Mater Misericordiae Private Hospital	James O'Neill
Mater Misericordiae University Hospital	Niall Mahon
Medizinische Hochschule Hannover	Christian Veltmann
Munich Grosshadern	Stefan Kääb
Na Homolce Hospital	Petr Neuzil
Northern General	Andreas Kyriacou
Nouvelles Cliniques Nantaises	Daniel Gras
Odense University Hospital	Jens Brock-Johansen
Ospedale Sacro Cuore Don Calabria	Giulio Molon
Papworth Hospital	Sharad Agarwal
Policlinico Casilino	Leonardo Calò
Rigshospitalet	Helen Høgh Petersen
Royal Hospital for Children	Karen McLeod
Royal Sussex	Jack McCready
Russells Hall	Craig Barr
Southampton General	Paul Roberts
St. Antonius	Lucas Boersma
Thoraxcentre Medisch Spectrum Twente	Marcoen Scholten
UH Schleswig-Holstein	Roland Tilz
UMC Groningen	Alexander Maass
Universitat - Herzzentrum	Christian Restle
Universitätsklinikum Muenster	Lars Eckhardt
University of Mannheim	Jùrgen Kuschyk
Yorkshire Heart Centre	Christopher Pepper

Supplementary Figure 1: Multivariable Predictors of Late Complications

Variable	Hazard Ratio	P-value
Valve Surgery vs no Valve Surgery	5.77 (2.22-15.03)	.0003
Cardiac Arrest vs no Cardiac Arrest	2.70 (1.24-5.91)	.013
Conversion Testing (CT) within 30 Days vs no CT within 30 days	0.26 (0.09-0.79)	.017
AF vs no AF	0.26 (0.08-0.90)	.034
Brugada vs no Brugada	3.69 (0.99-13.80)	.052
ARVC vs no ARVC	4.55 (0.95-21.85)	.059
BMI (per 1 kg/m²)*	1.05 (1.00-1.11)	.062
LVEF ≤ 35% vs LVEF > 35%	1.81 (0.80-4.10)	.16

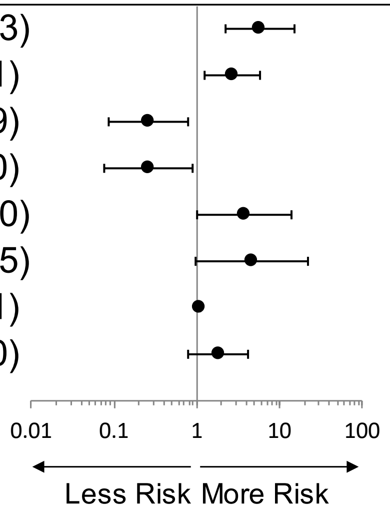

*continuous variable

Supplementary Figure 2: IAS Free Rate Years 2-5 after IAS Year 1

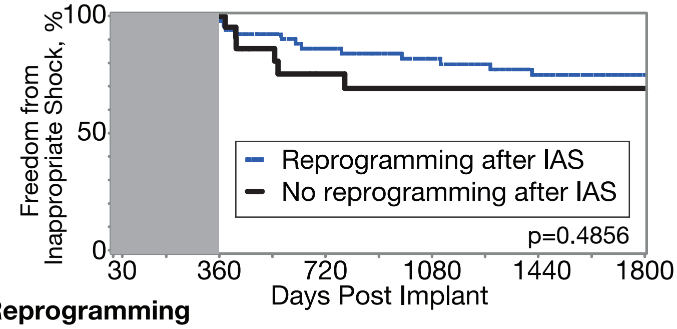

**Reprogramming**					
N at Risk	53	41	36	32	26
K-M Rate, %	100.0	86.3	82.0	75.1	75.1
**No Reprogramming**					
N at Risk	22	12	11	11	9
K-M Rate, %	100.0	75.6	69.3	69.3	69.3

Supplementary Figure 3: Time to therapy, Appropriate Episodes

Discrete Episodes

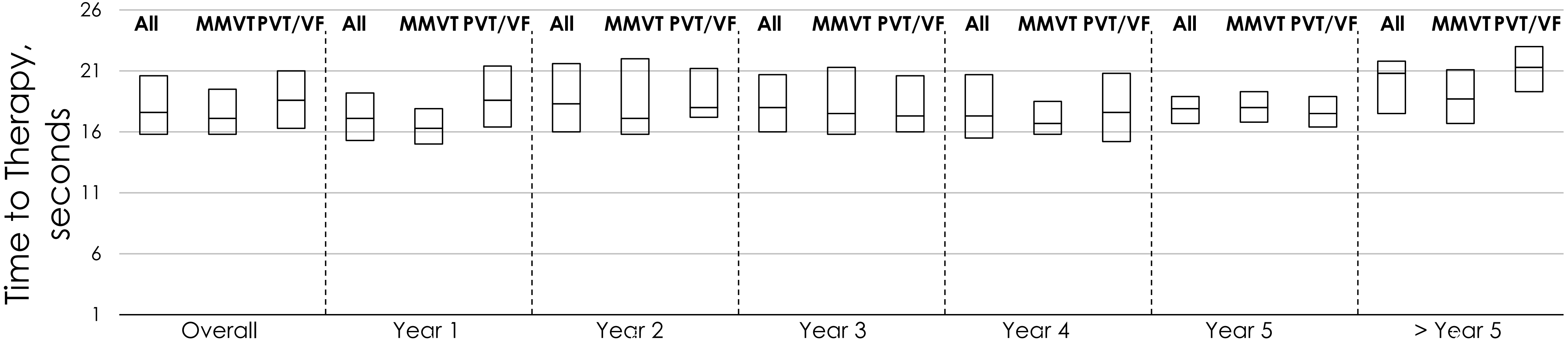

	Overall			Year 1			Year 2			Year 3			Year 4			Year 5			> Year 5		
	All	MMVT	PVT/VF	All	MMVT	PVT/VF	All	MMVT	PVT/VF	All	MMVT	PVT/VF	All	MMVT	PVT/VF	All	MMVT	PVT/VF	All	MMVT	PVT/VF
**Subjects**	146	86	81	58	39	26	38	17	25	40	23	18	35	18	18	23	15	8	14	8	7
**Episodes**	310	161	149	103	54	49	56	24	32	51	28	23	49	23	26	31	23	8	20	9	11
**Median TTT, s**	17.6	17.1	18.6	17.1	16.3	18.6	18.3	17.1	18.0	18.0	17.5	17.3	17.3	16.7	17.6	17.9	18.0	17.5	20.8	18.7	21.3

- Year p=0.054
- Rhythm type p=0.09
- Year x rhythm type p=0.25

Storm Episodes

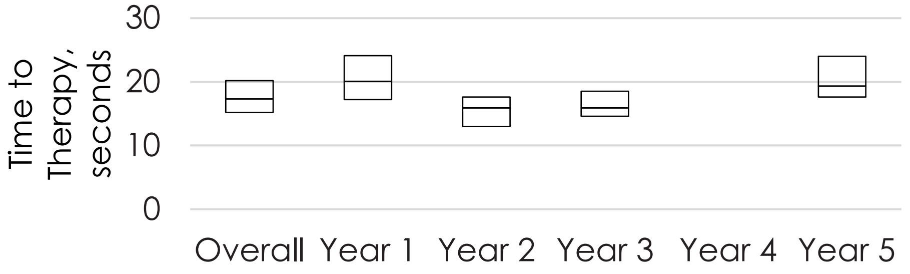

	Overall	Year 1	Year 2	Year 3	Year 4	Year 5
**Subjects**	18	6	7	4	0	2
**Storm Events**	21	7	8	4	0	2
**Episodes**	103	25	39	16	0	23
**Median TTT, s**	17.3	20.1	15.9	15.9	N/A	19.3

Year p=0.32
Rhythm type p=0.14
Year x rhythm type p =0.14

8 episodes from 7 patients with time to therapy >30 s

- 3 MMVT: near rate cutoff or discrimination error
- 5 PVT: the only one over 40 s had noise on signal. Others due to undersensing and some noise.

Supplementary Figure 4: Cause of Death

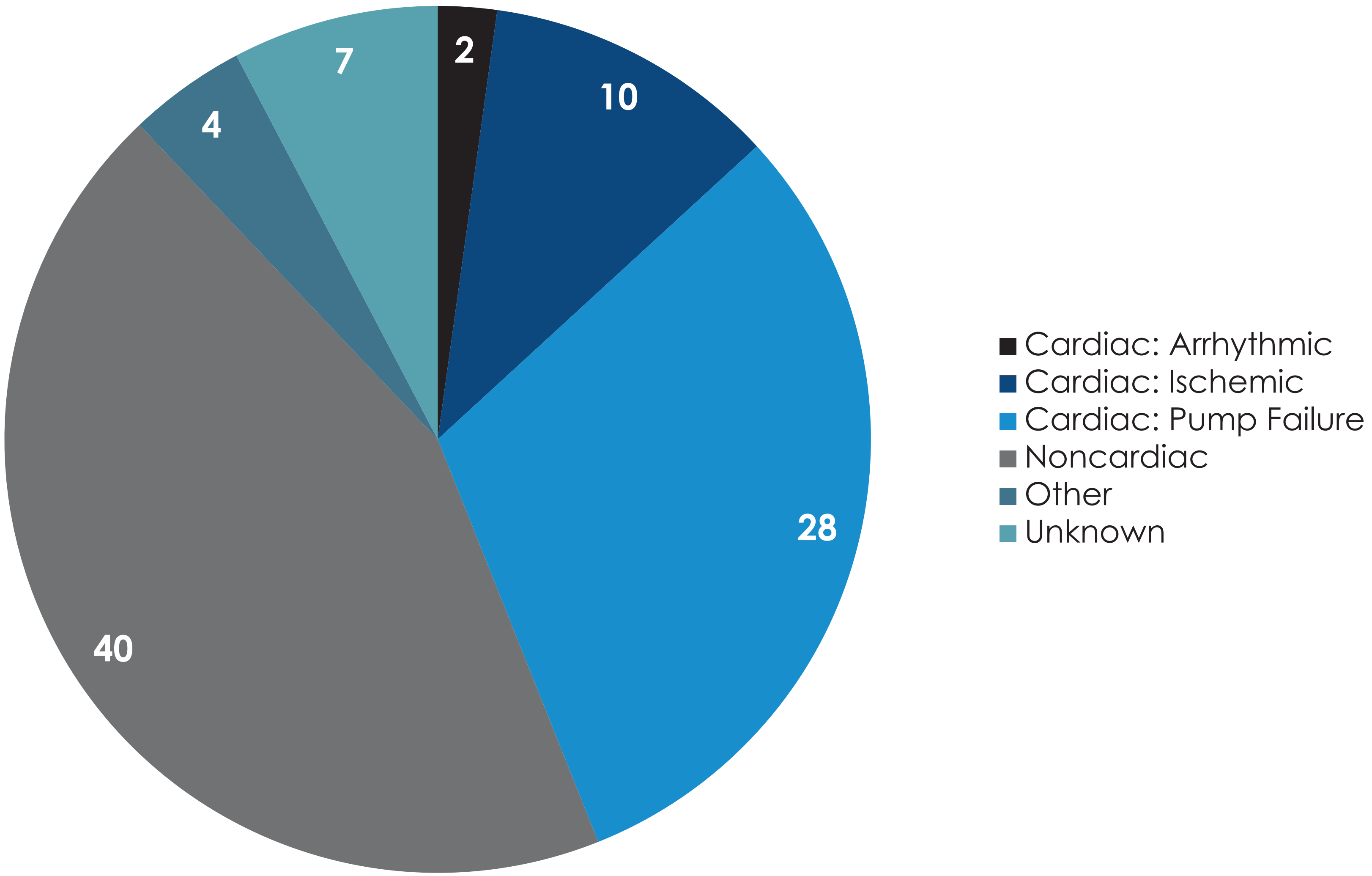

Supplement: ehab921_Supplementary_Data [file ehab921_supplementary_data.pdf]
